# The role of metabolic ecosystem in cancer progression — metabolic plasticity and mTOR hyperactivity in tumor tissues

**DOI:** 10.1007/s10555-021-10006-2

**Published:** 2022-01-14

**Authors:** Anna Sebestyén, Titanilla Dankó, Dániel Sztankovics, Dorottya Moldvai, Regina Raffay, Catherine Cervi, Ildikó Krencz, Viktória Zsiros, András Jeney, Gábor Petővári

**Affiliations:** 1 1St Department of Pathology and Experimental Cancer Research, Semmelweis University, Üllői út 26, 1085 Budapest, Hungary; 2 Department of Anatomy, Histology and Embryology, Semmelweis University, Tűzoltó utca 58, 1094 Budapest, Hungary

**Keywords:** Metabolic heterogeneity, Metabolic plasticity, Metabolic phenotypes, mTOR hyperactivity, Cancer

## Abstract

Despite advancements in cancer management, tumor relapse and metastasis are associated with poor outcomes in many cancers. Over the past decade, oncogene-driven carcinogenesis, dysregulated cellular signaling networks, dynamic changes in the tissue microenvironment, epithelial-mesenchymal transitions, protein expression within regulatory pathways, and their part in tumor progression are described in several studies. However, the complexity of metabolic enzyme expression is considerably under evaluated. Alterations in cellular metabolism determine the individual phenotype and behavior of cells, which is a well-recognized hallmark of cancer progression, especially in the adaptation mechanisms underlying therapy resistance. In metabolic symbiosis, cells compete, communicate, and even feed each other, supervised by tumor cells. Metabolic reprogramming forms a unique fingerprint for each tumor tissue, depending on the cellular content and genetic, epigenetic, and microenvironmental alterations of the developing cancer. Based on its sensing and effector functions, the mechanistic target of rapamycin (mTOR) kinase is considered the master regulator of metabolic adaptation. Moreover, mTOR kinase hyperactivity is associated with poor prognosis in various tumor types. *In situ* metabolic phenotyping in recent studies highlights the importance of metabolic plasticity, mTOR hyperactivity, and their role in tumor progression. In this review, we update recent developments in metabolic phenotyping of the cancer ecosystem, metabolic symbiosis, and plasticity which could provide new research directions in tumor biology. In addition, we suggest pathomorphological and analytical studies relating to metabolic alterations, mTOR activity, and their associations which are necessary to improve understanding of tumor heterogeneity and expand the therapeutic management of cancer.

## Introduction

Metabolic alterations and bioenergetic adaptation mechanisms are essential components of the metabolic ecosystem and play a key role in cancer progression. In the past two decades, considerable advancements have been achieved in this field of research. *Warburg* and *Minami* were the first to describe altered tumor metabolism in 1923 [1]. Approximately one century later, metabolic symbiosis of tumor tissues, including alterations within the tumor microenvironment, were highlighted among the main hallmarks of cancer [2]. The term “Warburg effect” was coined in the 1970s, referring to rapid glucose uptake and high-rate lactate secretion into the tumor microenvironment (increased acidification). This fundamental feature of the anabolic processes is necessary for cancer progression and tumor cell proliferation, where the conversion of glucose to lactate provides a large pool of glycolytic metabolites and fuels the pentose phosphate and other macromolecule synthetic pathways. Glucose utilization is influenced by the interactions of cancer cells and their surrounding microenvironment, which further impacts tumor evolution (progression, adaptation mechanisms, therapy responses, and metastasis). The heterogeneity and the hierarchy of tumor tissues determine the complex ecosystem [3]; moreover, metabolic heterogeneity and metabolic symbiosis influence the homeostasis of the whole organ.

Proliferating and inflammatory cells invade the microenvironment causing continual destruction of non-tumorous cells, contributing to the development of cancer within actively participating stromal elements. Tumor cells reorganize the cellular milieu (fibroblasts, immune, mesenchymal, and other non-tumorous cells, etc.), coordinating the evolution of tumor tissue [4]. The extracellular matrix (ECM) provides nutrients and building blocks for the proliferating tumor cells. Metabolic “waste” such as metabolites and activating cellular elements accumulate and play a role in the paracrine regulatory functions of surrounding cells. This activity results in ECM remodeling via signaling mechanisms in all cells or related matrix elements [5, 6]. Considering tissue heterogeneity, parts of the cell population may vary between well/moderately/poorly differentiated morphology. Furthermore, the mutation and protein expression patterns of tumor tissue must also be considered by pathologists and clinicians during differential diagnosis. Consequently, these factors influence the therapeutic management of cancer. However, the prognostic capacity of the initial primary biopsy is limited and does not represent the real complexity and alterations of relapsed or metastatic tissues (following therapeutic intervention). Genetic and epigenetic regulation and protein expression of the developing tumor can be altered by several factors, e.g., inflammation and hypoxia (inefficient angiogenesis). Therefore, it is essential to examine cancer on a cellular, phenotypic, and genomic level, to greater characterize these features and understand their clinical relevance. There are many histopathological reviews about tumor mapping and stromal and immune cells within their ECM structure. *In vivo* molecular imaging with positron emission tomography (PET) and magnetic resonance imaging (MRI) allow *in situ* morphological analysis and emphasize the significance of metabolic and cellular heterogeneity. These results suggest that multicellular symbiosis has complex, competitive advantages in metabolic tumor ecosystems supporting the high adaptation potential (plasticity) for malignant cells during tumor growth, resistance, and metastasis. Cancer can be considered as a dynamic ecosystem where tumor cells cooperate among each other and host neighboring non-tumorous cells within their microenvironment. Understanding these interactions at tissue level modifies and develops the therapeutic management of cancer, with the application of targeted and immune checkpoint inhibitors combined with conventional therapies. Promising ongoing and upcoming investigations expand the availability of actionable targets and targeted therapies against cancer.

**Fig. 1:**
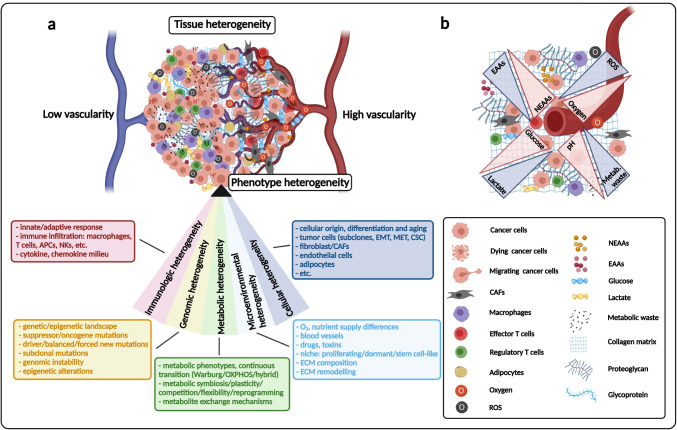
Main participants of tumor heterogeneity and the complex cellular milieu in tumor tissue. **a** Many different factors influence tumor development in correlation with the hallmarks of cancer. In the last century, terminology relating to tumor tissue heterogeneity was introduced. Each type of heterogeneity has been extensively studied; based on these, the figure describes the associated areas of cancer research. The summarized “heterogeneity section” consists of subcategories including cellular elements, genetic diversity, immune, microenvironmental, and metabolic alterations of the tumor (these main elements and their contributors are indicated). The shown heterogeneities in the sector contribute to the individual phenotypes, which constitute the complex ecosystem (with tumor and non-tumorous cells in tissue structure). Stress and starving conditions influence tissue heterogeneity and cellular proliferation, differentiation, and survival mechanisms. Unfortunately, evolutionary mechanisms may select highly aggressive cell populations in the developing ecosystem, contributing to disease relapse or metastasis in patients. (APCs, antigen-presenting cells; NKs, natural killer cells; EMT, epithelial-mesenchymal transition; MET, mesenchymal-epithelial transition; CSC, cancer stem cell; CAFs, cancer-associated fibroblasts; ECM, extracellular matrix). **b** Metabolic complexity of the tumor tissue in correlation with vascularization. The altered concentrations of glucose-lactate, oxygen-ROS (reactive oxygen species), H^+^ (pH)-metabolic waste, and amino acids (essential and nonessential amino acids — EAAs/NEAAs); and their altering gradients are labeled with blue/red triangles. The labeling of different cellular elements and other matrix components are also indicated

In the middle of the last century, it was stated that different cell subpopulations exist in “monoclonal cancer” [7–9]. *Fidler* confirmed tumor heterogeneity (intracellular heterogeneity) using B16 melanoma cell lines as an *in vivo* experimental model in 1978 [10]. Accordingly, it was demonstrated that only a few highly metastatic cells were present in the original B16 tumor population. This was verified with the use of an adapted classical fluctuation origin test, by *Luria* and *Delbrück*. Considerable variations in the number and sites of pulmonary metastases were detected using different cell “clones.” In the 1980s, *Miller* highlighted the phrase *intratumoral heterogeneity* in Cancer and Metastasis Reviews [11] and finally, *Miller* and *Heppner* coined the terms *tumor heterogeneity* and intratumoral heterogeneity in subsequent publications [12, 13]. Currently, several types of tissue heterogeneities can be distinguished, e.g., cellular, environmental, genomic, epigenomic, phenotypic, and metabolic heterogeneity. All of these have influencing effects on tissue structure, resulting in the development of tissue heterogeneity, diversity, and plasticity of cancer (Fig. 1). This complex network (ecosystem) serves as the basis for developing tumors [3].

Environmental adaptation, metabolic activity, and rewiring are critical for maintaining the survival and growth of the cancer cell population. Furthermore, these processes have central regulatory roles in tumor progression and therapeutic failure. Heterogeneous metabolic activity in tumors is characterized based on morphological and pathological examinations, tissue distributions of different tracers via functional imaging analysis, and the staining patterns of several metabolic transporters and enzyme expressions. *Metabolic heterogeneity* and its consequences (*metabolic plasticity*) also affect tumor cell survival and growth *in vivo*. Metabolic plasticity occurs when the “swamp occupies the healthy organized tissue” and induces metabolic collapse and the final death of the host organism. In this way, the metabolic plasticity of the tumor tissue ensures survival of the tumor population at all costs. Therefore, better characterization of the metabolic phenotypes and a deeper understanding of the metabolic dependencies of tumor and stromal elements and their network could improve the therapeutic management of cancer using novel or already available antimetabolic drugs. Finally, mapping metabolic heterogeneity (at tissue level) is necessary via analytical and pathomorphological examinations. This study aims to contribute to this growing area of research by exploring the available publications and summarizing the upcoming developments in metabolic heterogeneity and their implications in a variety of cancers. Recent developments in multicellular model systems and morphological and imaging studies have improved the understanding of the complexity of tissue heterogeneity from a metabolism perspective. Based on these findings, a multidisciplinary approach could improve personalized therapies and patient outcomes.

## Metabolic consequences of frequent genetic mutations

*RAS*, *p53*, and *MYC* are common driver mutations (mutated oncogene/tumor suppressor and amplified genes) that cause regulatory alterations, affecting the cancer metabolism directly and indirectly [14]. MYC regulates metabolic enzymes, which orchestrate metabolic homeostasis, and plays a critical role in tumorigenesis, proliferation, and cell growth. This transcription factor also regulates several metabolic enzymes and transporters (e.g., phosphofructokinase 1 — PFK1, hexokinase — HK, lactate dehydrogenase A— LDHA, glutaminase — GLS, ATP citrate lyase — ACLY, amino acid transporter solute carrier family 1 member 2 — SLC1A5, glucose transporter 1 — GLUT1, monocarboxylate transporter 4 — MCT4). Additionally, *MYC* is involved in glucose/glutamine sensing and mTOR-dependent Akt functions, which regulate the bioenergetic balance of tumor cell growth and survival [15]. Several studies underline the importance of oncogenic Ras-mediated metabolic shifts in tumor progression, which could influence and enhance the *MYC*-regulated cellular events via activation of the Raf/Mek/Erk pathways. Moreover, *RAS* promotes anabolic mechanisms involved in fueling mitochondrial ATP generation. Increased autophagy is observed in *RAS*-driven tumor cells. Additionally, extracellular albumin and lipid consumption can be increased through macropinocytosis [16, 17]. These mechanisms help to utilize both extra- and intracellular resources and recycle many different metabolites via anabolic shifts to generate new cells in the growing tumor population. These *RAS*-driven alterations, non-oxidative pentose phosphate pathways (PPP), glutamine/glutathione metabolism–mediated maintaining of redox homeostasis, and reactive oxygen species (ROS) detoxification are important in cancer progression [18]. In addition, p53 regulates cellular metabolism by multiple mechanisms and is often referred to as the guardian of the genome and cellular integrity. It can be activated in several cellular stress situations, including starving conditions, e.g., energy and nutrient depletion. In correlation with these, AMPK activates p53 either directly or through other mechanisms [19–21].

Consequently, glycolysis is reduced, and mitochondrial respiration is increased directly by *TP53*-induced glycolysis and apoptosis regulator (TIGAR). In turn, this reduces the activity of Akt/mTOR, NF-κB, PPP, glucose transporters, and the expression of glycolytic enzymes (PFKs; pyruvate dehydrogenase kinase 2 — PDH2) and increases PDH activation [22, 23]. In parallel, mitochondrial oxidative phosphorylation is positively regulated by p53 in cells which facilitate pyruvate–acetyl-CoA conversion and mitochondrial glutaminolysis by GLS2, promoting mitochondrial oxidative processes and enhancing glutathione production, thus maintaining redox homeostasis [24]. Furthermore, it was described that p53 can directly bind and depress sterol regulatory element-binding transcription factor 1 (SREBP1), inhibit lipid synthesis, and enhance fatty acid (FA) oxidation in cells [23]. Moreover, mutant p53 could have additional roles in metabolic reprogramming with active participation. It was reported that these mutant proteins could bind and activate transcription factors, including SREBP1/2 and ETS2, which upregulate both lipid and nucleotide biosynthesis in malignant cells [25]. These results highlight *TP53* mutation and its part in the loss of unfavorable regulatory functions. However, they could also have a direct gain-of-function effect within the metabolic regulatory network and mutation hotspot in a cell type–dependent manner. Beyond the most frequently mutated driver genes, several other mutations also have metabolic consequences during tumorigenesis. Dynamic tissue remodeling, signaling alterations due to oncogenic mutations, and their metabolic products have been widely investigated and mapped.

During tumor progression, especially in metastatic processes, cells undergo an epithelial-mesenchymal transition (EMT); this process has unique demands and requires metabolic rewiring mechanisms. *Mutation-dependent activation of Wnt/-catenin signaling* is characteristic for mesenchymal transformation in epithelial cells, which lose their differentiated phenotype. For example, mutations in the adenomatous polyposis coli gene and altered Wnt signaling–related events are associated with familial adenomatous polyposis in colonic tumors [26]. Crosstalking between other signaling network kinases (as MAPK, MEK1, and LKB1) and oncogenic signaling pathways including Wnt, transforming growth factor-β (TGFβ), NOTCH, and JAK/STAT has several effects in tumor progression, especially in EMT. These signaling alterations induce the overexpression of SNAIL1-2/ZEB1-2/TWIST transcription factors (as a consequence of EMT signaling activation) and downregulate the expression of several glycolytic enzymes. Additionally, this promotes glutamine and asparagine metabolism. These genetic alterations, which result in Wnt/TGFβ/NOTCH signaling hyperactivity or other EMT-forcing events, alter the metabolic activity of cancer cells. The concomitant adaptation mechanisms initiate the anchorage-independent migration, the survival of cancer cells in particular conditions, and even the metastasis formation in the whole body [27]. In contrast with a proliferating tumor mass, metastatic cells have a “slow life,” which is characterized by their catabolism, resistance to cell death, generation of ATP from oxidative phosphorylation (OXPHOS), quenching ROS, and adaptation mechanisms using alternative energy sources such as autophagy rather than external sources [28–30].

It has been suggested that other relatively frequent mutations like *EGFR* and *PI3KCA* activations correlate with the *in situ* proliferation and metabolic alterations (e.g., in non-small cell lung cancer — NSCLCs and breast carcinomas). A positive correlation was found among PET-CT SUVmax, *EGFR*, glycolytic activity, and *PI3KCA* mutation status in NSCLCs and breast carcinomas [31, 32]. These results support the conclusion that not only *TP53* and *KRAS* but also receptor *tyrosine kinase pathway mutations* are responsible for the metabolic switch to increased glycolysis and reduced OXPHOS [32–34]. Additionally, epigenetic alterations (e.g., hypermethylation) and *PTEN* suppression result in *PDK1/MYC*-dependent Akt/mTOR activation. These are commonly found in targeted receptor inhibitor-resistant carcinoma cells [35]. The loss of other negative regulators in these signaling pathways and their downstream consequences may be targetable with mTOR kinase inhibitors (e.g., in cancers harboring TSC1/2 and PTEN germline or somatic mutations).

Metabolic rewiring in cancer is associated with oncogenic alterations, which have been extensively examined in the past few years. The data summarized above demonstrates how the different oncogenic mutations and their metabolic consequences affect cancer metabolism. Finally, these advancements may improve understanding of oncogenic mutations and expand the therapeutic management with the addition of antimetabolic drugs to combined therapies.

## Increased amount of oncometabolites

**Fig. 2:**
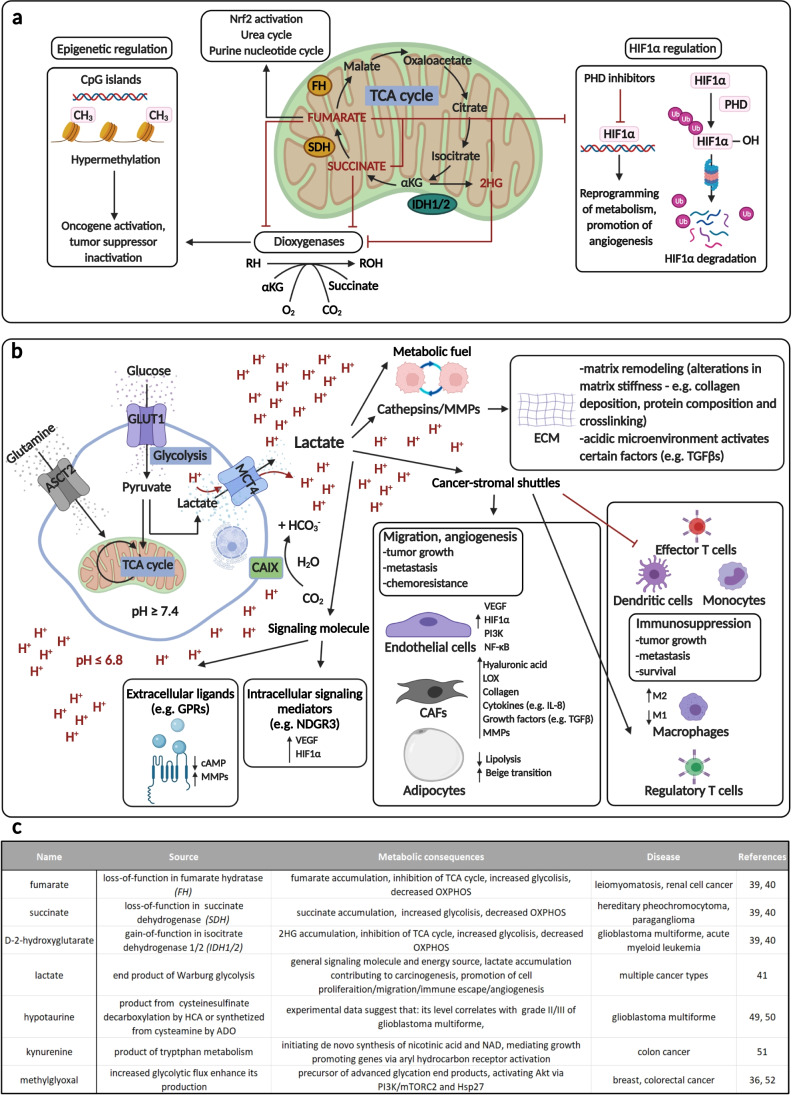
The tumor-promoting effects of oncometabolites. **a** The classical oncometabolites (fumarate, succinate, D-2-hydroxyglutarate — 2HG) and their epigenetic and angiogenic effects are shown in the figure. The accumulation of these oncometabolites inhibit prolyl hydroxylases and stabilize HIF1α (causing hypoxia), and they have a direct regulatory role by competitively inhibiting dioxygenases, influencing promoter methylation — activation/inactivation of oncogenes and tumor suppressor genes, respectively. **b** Lactate (as an oncometabolite) causes an acidic microenvironment, which aids tumor progression. Tumor and stromal cells produce lactate in correlation with blood vessel distance and tumorigenic alterations (e.g., oncogene-driven pseudohypoxia). Lactate and decreased pH have many tumor promoter functions: inhibition of antitumor immune effector cells, induction of therapy resistance, and sourcing of alternative nutrient supply for highly oxygenated normal and malignant cells. The acidic microenvironment also causes cancer-associated fibroblasts (CAFs) to produce growth factors, cytokines, feed tumor cells (TCA replenishing metabolites and the use of amino acids as nutrients), and matrix degradation. Additionally, the low pH could play a role in cytokine and enzyme activation, assisting with tumor cell adaptation, matrix remodeling, and tumor cell migration, as part of immunosuppressive and tumor-promoting niches. **c** The names, sources, metabolic contributions, and the relevance of traditional and non-traditional oncometabolites in various cancers

Germline or somatic mutations in metabolic genes can cause “inborn” errors in metabolism and increased cancer risk [36, 37]. Mutations and loss of function of certain metabolic enzymes (fumarate hydratase — FH and succinate dehydrogenase — SDH) in the tricarboxylic acid (TCA) cycle are described as precursors of rare inherited and renal cancers. In 2008, isocitrate dehydrogenase (IDH1/2) mutations and the accumulation of D-2-hydroxyglutarate (2HG) were discovered to have a tumorigenic role in gliomas [38]. “Oncometabolites” were distinguished from metabolic toxins and other small metabolites based on their promoting role in malignant transformation, tumor growth, and progression [39]. There are many observations regarding tumor cell–driven metabolic by-products in high intra- or extracellular concentrations as well as their role in propagating and promoting tumor growth. Due to their structural similarities, *fumarate*, *succinate*, and *2HG* can competitively inhibit alpha-ketoglutarate (αKG)–dependent dioxygenases (increasing HIF1α, pseudohypoxia). These oncometabolites also competitively inhibit epigenetic regulatory proteins (histone lysine demethylases — KDMs, ten-eleven translocation — TET, and 5-methylcytosine hydroxylases) by influencing methylation and acetylation functions [39, 40]. Furthermore, these mutations and altered enzyme functions cause mitochondrial failures, resulting in increased ROS with several consequences (Fig. 2.).

According to the literature, the most frequent tumorigenic alterations increase *lactate* production, causing acidification in tumor tissue; this is a well-known metabolic consequence of fast proliferating tumor cells. Based on these studies, lactate is not only a by-product but an important oncometabolite in tumor homeostasis [41]. In summary, elevated lactate concentrations in tumor tissues and blood are correlated with a high risk of cancer progression, metastasis, and mortality.

Lactate has a significant role in metabolic symbiosis; it can be used and oxidized by highly oxygenated cells. In addition, lactate is a signaling molecule responsible for further extra- and intracellular effects in the microenvironment. Elevated lactate levels influence the production of several growth factors by fibroblasts, endothelial cells, and adipocytes (HGF, ILs, TGFβ, IFNs, FGFs, VEGF, etc.). Consequently, this can induce the expression of some regulatory proteins, metabolites, microRNAs (miRs), and their exosomal transport [4, 42, 43]. Lactate can bind NDGR3, preventing association with PHD2, and stabilize HIF1α by inhibiting prolyl hydroxylase (PHD) activity [44]. Moreover, proton-sensitive lactate sensors (GPR4/65/68/132) in the acidic tissue microenvironment can activate intracellular Ca^2+^-cAMP-ROS and the related MAPK pathways, leading to increased matrix metalloproteinase (MMP) expression and stimulation of EMT in the tumor. Additionally, acidic pH induces the proteolysis of ECM elements and activates MMPs and cathepsins [45]. Acidic conditions in the microenvironment cause redistribution of lysosomes to the cell periphery, e.g., secretion of cathepsin B to the microenvironment. These factors contribute to tissue remodeling and alteration of tumor cell polarity and adhesion properties [46, 47]. Lactate and the acidic microenvironment negatively influence the immune response, affecting tumor-infiltrating immune cells in many ways. These microenvironmental changes induce immunosuppressive functions via inhibition of immune effector cells; reduction of natural killer, cytotoxic T cells infiltration, and monocyte-macrophage differentiation (inducing a shift to M2 macrophages), impairment of antigen presentation, and supporting of regulatory T cells. These all contribute to the tumor immune escape mechanism [37, 41, 48]. Finally, all these alterations promote survival, motility, migration, and invasion of cancer cells (Fig. 2).

Besides the classical oncometabolites (fumarate, succinate, 2HG), lactate is the most important and well-studied oncometabolite. In recent metabolomic studies, several putative oncometabolites have been reported. There is abundant room for further progress in determining additional oncometabolites and their tumorigenic effect in different cancers. For example, in gliomas, increased levels of *hypotaurine* are strongly correlated with the tumor grade. Additionally, homocysteic acid (an inhibitor of cysteine sulfinic acid decarboxylase — hypotaurine production) can inhibit the proliferation of glioblastoma cell lines, and some *in vivo* studies have confirmed that hypotaurine could be a targetable oncometabolite in glioblastomas [49, 50]. *Kynurenine* is known to relate to *de novo* nicotinic acid and NAD synthesis. Based on these findings, it is thought to have an oncometabolite function, e.g., in colon cancers [51]. Additionally, *methylglyoxal* involves the formation of advanced glycation end-products, which could have tumor-promoting effects. In conclusion, using the term oncometabolites for methylglyoxal and other potential metabolites requires further experimental and clinical investigations [52] (Fig. 2).

## ECM as a metabolic niche in tissue microenvironment

Alongside tumor and non-tumorous cells, the functions of the extracellular matrix and their role in carcinogenesis and cancer progression are extensively reported in the literature. As a part of metabolic symbiosis, *tumor microenvironment heterogeneity* is a proposed hallmark of cancer metabolism and is responsible for the specific metabolic niche in the tumor mass [2, 5, 6]. Consequently, this affects tumor growth in a complex manner [4].

Similarly, to wound healing, the growing tumor also influences stromal elements and matrix proteins and provides nutrients (proteins, amino acids, lipids, and their building blocks). As elements of tissue remodeling, cancer cells reorganize the surrounding cellular milieu, which promotes both tissue regeneration and the production of several additional factors (e.g., cytokines and growth factors). The alterations in danger-associated molecular patterns propagate the differentiation of myofibroblast-like to cancer-associated fibroblasts (CAFs). Tumor cell–derived metabolic shifts directly affect the tumor type–dependent metabolites as well as the cellular nutrient demand and can completely exhaust the matrix and release metabolic products into the ECM [53] (Fig. 1).

The hypoxic or pseudohypoxic changes induce the Warburg effect; therefore, the concentration of glucose, lactate, and other metabolic intermediates (e.g., carbohydrates, ribose, specific lipids, FAs, acetate, and amino acids) can vary in cancer microenvironments. Increased lactate production and/or O_2_ consumption resulting CO_2_ release and their transport mechanisms (using MCTs and carbonic anhydrase IX (CAIX) transporters) also contribute to lowered pH in the tumor mass [54, 55].

Glucose and amino acids are the primary carbon sources for proliferating cells in tumor tissues [56–59]. However, glutamine utilization and its replenishing effect (anaplerosis) in the TCA cycle can support the tumor growth/survival processes under hypoxic conditions [60]. While glutamine uptake maintains nucleotide biosynthesis, glutathione plays a role in the exchange of nonessential amino acids. Therefore, glutamine, serine, and cysteine concentrations are reduced in the ECM. Other utilization processes can be propagated in case of inadequate vascularization and reduce the levels of building block metabolites within the ECM. Acetate- and/or citrate-derived acetyl-CoA could be used in lipid/FA biosynthesis, especially in RAS-transformed and/or acetyl-CoA synthase 2 (ACSS2) overexpressing cells [61]. As mentioned previously, growing tumors are similar to dysregulated wound healing based on their high-rate glucose consumption, lactate production, and depletion of extracellular glutamine [4]. To speed up glucose uptake and support catabolism, tumor cells have advantages due to their oncogenic alterations. The studies presented thus far provide evidence that the glucose concentration is typically lowered by about 90% in tissue fluids during intensive tumor proliferation [62]. Immune effector cells also have high glucose demands; therefore, lowered glucose impairs their functions (e.g., IFNγ production, Th1 differentiation) [63]. Based on these factors, the available glucose concentration could be an important metabolic checkpoint in the downregulation of antitumor immune response (in addition to increasing the lactate levels). The importance of sufficient nutrient sources was described in PD-L1 immune checkpoint therapy. In correlation with this, GLUT1 expression was found to be upregulated in effector T cell population, forcing glucose uptake from tumor microenvironment [63]. It was also described that the efficacy of adaptive T cell therapy was lower in highly glycolytic tumors [64].

Moreover, starving conditions and lowered ATP level induce autophagy of stromal fibroblasts to replenish nutrition for the ECM (amino acids and nonessential amino acids) via the activation of AMPK and subsequent inhibition of the mTOR pathway [65]. Low glucose concentration decreases PFK1 and the proliferation and migration of endothelial cells, and, as a further consequence, this inhibits the vascularization and nutrient supply in the whole tumor mass triggering continuous tissue regeneration. These also contribute to the lactate accumulation in the ECM [41].

Based on these findings, the metabolic plasticity and symbiosis among different cellular elements and their metabolic subtypes highlight the importance of glucose and lactate – as metabolic substrates in the tumor microenvironment [66]. The lactate concentration changes and the appearance of pH alterations are being studied in greater detail, especially regarding their contribution to tumor progression and drug resistance [48].

Amino acids and their uptake could have critical importance in tumor proliferation [67]. Glutamine consumption could be essential in promoting intensive proliferation and refueling of the TCA cycle. Moreover, similar to serine, glutamine is also important for nucleotide synthesis and maintaining redox homeostasis through the production and exchange of glutathione [68]. In amino acid– and nutrient-depleted conditions, the increased expression of transporters and oncogene-driven alteration mechanisms induce macropinocytosis of ECM proteins to support rapidly growing tumor cell populations [69]. The induced autophagy in CAFs can provide other amino acids and dipeptides. Additionally, adipocytes can provide FAs and other nutrients under these conditions. These effects may have a supporting role in cancer cell proliferation [70]. Many cancer types have described the correlation between lipid droplet (LD) accumulation and chemoresistance [71]. Furthermore, the exogenous lipids and obesity-related factors could influence metastasis [72, 73].

It is also known that other major molecular components of the ECM (collagen, laminin, fibronectin, proteoglycans, and elastin) may be degraded by acidosis-activated proteases and MMPs. These ECM-released nutrients could impact the metabolic regulation of tumor growth. Moreover, negatively charged glycose-amino-glycans within the ECM can alter proton and cytokine distribution, playing a role in re-localization in the pre-metastatic niche and metastasis progression. It was also suggested that cell surface proteoglycans can bind low-density lipoproteins and help in their internalization [74, 75]. Exosomes enriched in proteoglycan-bounded lipoproteins could influence cellular communication and vesicle cargo [76]. In addition, nucleic acids, protein, and exosomal metabolites could influence the invasive potential of cancer cells (e.g., melanoma cells) [77].

In summary, these alterations adjust metabolic symbiosis (many cells and biomolecules compete, communicate, and even feed each other as dominated by tumor cells) causing metabolic rewiring of tumor cells, influence crosstalk between tumor and non-tumorous cells, increase tumor tissue heterogeneity, and maintain the homeostasis of the organ/whole organism [78, 79].

## Dynamic EMT-MET transitions and their metabolic consequences (metabolic plasticity) in cancer tissues; cancer stem cells, dormant cells

Differentiation, dedifferentiation, transdifferentiation, and stemness are central elements of organ development, inflammation, tissue regeneration, and repair. These underline the importance of phenotypic plasticity and its contribution to cancer initiation, development, and progression. Previously, transdifferentiations were believed to be limited to inferior vertebrates (e.g., amphibians); however, it is now understood that mammalian cells can dedifferentiate and transdifferentiate. Several transcription factors could reverse the actual cellular phenotype and initiate the reprogramming of human cells, e.g., fibroblasts to cardiomyocytes, neurons, among other cell types [80]. MyoD, OCT4, SOX2, KLF4, and Myc reprogram mammalian fibroblasts to an embryonic-like state [81–83]. The related experimental results highlighted the potential role of cellular plasticity in tumorigenesis. Based on the studies from the last decade, it was accepted that tumors can hijack the differentiation programs of the original cells [84]. The activation of EMT (and its reverse process; mesenchymal-to-epithelial transition — MET) drives differentiation during tumor initiation and progression [85, 86]. In these transitions, cells gain several features such as plasticity and some stem cell–like properties. Further consequences include loss of polarity and adherence to the basement membrane in epithelial cells, reorganization of their cytoskeletal structure, expression of specific mesenchymal cell markers, as well as cellular migration and renewal.

Recent studies on colon and skin carcinogenesis have identified several transitional stages in tumor initiation and progression [87]. Cells within the heterogeneous tumor tissue show different stages of EMT and diverse *intermediate (hybrid) phenotypes* during epithelial and mesenchymal transition states. It was also described that these different subpopulations had altered tumorigenic properties, colony formation, and metastatic capacity *in vivo*. The EMT phenotype and mesenchymal characteristics of circulating tumor cells (CTCs) correlate with their metastatic potential and have prognostic significance for cancer patients [88]. Several results suggest the role of EMT in dedifferentiation and altered transcriptional regulatory network in the loss of therapy sensitivity [89, 90].

It was proposed that the amount of CSCs correlates with the tumorigenicity of the cell population (e.g., aggressiveness during *in vivo* xenotransplantation) [91]. *CSCs and dormant cancer cells* are often considered the same; however, these are definitively not interchangeable terms. CSCs have special characteristics, including self-renewal features together with their special differentiation state. At the same point of cell maturation, dormant cancer cells are “similar” to their active tumor cell pair [92]. In addition, dormant tumor cells and their tumor counterparts can switch back and forth in correlation with their functions and metabolic states during tumor evolution. To add this complexity, the CSC population is inhomogeneous, and cancer dormancy can have several meanings, depending on the definition used. “Cancer dormancy” was originally used in the clinical setting corresponding with “tumor mass dormancy,” when the tumor was at undetectable level with a balanced tumor cell proliferation and death (*clinical dormancy*) [93–95]. Dormant cancer cells have different and frequently misunderstood meanings. Additionally, non-proliferating, quiescent cancer cells are in the G0-G1 phase at cell cycle arrest. Quiescence and dormancy are almost similar cell states that can be distinguished based on how they re-enter to the cell cycle. Quiescence, also referred to as G0, is a temporary pause of proliferation that will be resumed when conditions are favorable. On the other hand, dormancy is perceived as a deeper arrested state, suggesting that dormancy is more persistent than quiescence. In comparison to this, the stem cells are not arrested in the cell cycle and have a self-renewal (asymmetric division capacity).

The dormant and quiescent cells are different from slow-cycling or other CSCs. However, they have many similarities from a transcription factor expression point of view (e.g., overexpression of SOX2, NANOG, and NRF2). Both dormant cells and CSCs are rare in tumor tissue, and the presence of these cells could be a source of relapse and drug resistance. To characterize these cells, several markers can be used; SOX2, N-cadherin, and CD13 are usually expressed in both cell types. Additionally, there are some other intracellular and adhesion proteins which can help to distinguish these states; e.g., OCT4, increased RORγ transcription factors, ALDH1 enzyme, c-Met, EpCAM, CD44, CD133, CD24, ABCG2, ABC transporter expression, and the marker of cycling cells (Ki67) are characteristic for cancer stem cells. However, the expression pattern highly depends on the tumor type [96–98]. To select dormant cells, other marker sets are necessary, for example, increased expression of p21, p27, FBXW7, NR2F1, DEC2, activated p38, and inhibition of TGFβ1-2 and VEGF signaling, with decreased levels of ZEB1 [99]. The original “seed and soil” hypothesis underlines the regulatory role of the special microenvironmental niche in cellular differentiation (dedifferentiation, redifferentiation) and metastasis during tumor evolution.

In cancer development and progression, the above mentioned plasticity can be accounted for by tumorigenic mutations, environmental changes, or response to therapeutic interventions. Accordingly, the activation of EMT in cancer cells is closely related to stem cell–like and dormant cell phenotypes, which also participate in cellular plasticity. These all could contribute to tissue heterogeneity and help the adaptation of tumor cell population during extreme situations [79, 83]. EMT, cancer cell dedifferentiation, stemness, and dormancy require different metabolic activities. These states are connected with the altered sensitivity to microenvironmental conditions and are associated with tumor cell survival.

Metabolic adaptation depends on several factors such as the cell type, mutational profile (oncogenic alterations), and extracellular conditions. This emphasizes the individuality and complexity of each tumor and their development. Therefore, it is not surprising that papers published over the recent decade have many conflicting results about the metabolic characteristics of EMT, stemness, and dormancy.

EMT/MET alterations and their metabolic associations can be determined by transcriptomic and metabolic analyses in different tumors, where induced mitochondrial respiration and decreased Warburg glycolysis dependence in quiescent cells are shown [100]. Furthermore, the increased autophagy and lysosomal degradation as metabolic differences were also reported in many different cancer stem cells (e.g., pancreatic cancer and glioblastoma cells) [101, 102]. On the contrary, transcriptomic and experimental studies showed decreased mitochondrial OXPHOS enzyme expression and mitochondrial mass in different cancers (e.g., hepatocellular and renal cancers) in their dedifferentiated stem cell–like surviving tumor cells. These findings suggest that dormant and stem cell mechanisms are more complex, tumor type, and environmental condition–dependent [103].

A small population of aggressive, less differentiated CSCs with self-renewal capacity were reported to be responsible for therapy resistance, metastasis, and disease relapse. There are several contradictions regarding the metabolic phenotype of CSCs; depending on the stem cell enrichment method, isolation techniques, model systems, and definition of CSCs applied in a given study (characteristic marker sets of CSCs), for example, in lung, breast, and ovarian cancers and glioblastomas [100, 104]. The metabolic activity of normal stem cells relies on glycolysis; however, CSCs can use aerobic glycolysis or OXPHOS mechanisms depending on their tumor type and microenvironment (hypoxia, starvations) [105–107].

The original stem cell and CSC studies — the principal hypothesis — suggested that cellular metabolism controls stemness and CSCs could not be directly linked to a specific metabolic phenotype. Several studies demonstrated that metabolic rewiring is a glycolytic shift in CSCs, e.g., hepatocellular, colorectal carcinomas, osteosarcomas, and radioresistant nasopharyngeal carcinomas [104, 105, 108]. Surprisingly, the OXPHOS-dependent metabolism of CSCs is found in other tumor types (glioblastoma, pancreatic ductal adenocarcinoma, breast cancers, and AML). Moreover, increased nutrient utilization (such as glutamine or other amino acids, fatty acids, ketones) and alternative energy supplementation (especially the environment-independent survival benefits) are associated with tumor cell stemness [109–112]. One of the essential amino acid substrates in cancer cells is glutamine, and glutamine utilization could have particular importance in certain CSCs (e.g., breast and pancreatic cancers). Based on these results, the mitochondrial functions and alternative metabolic pathways (glutamine uptake and *de novo* synthetic capacity) play an essential role in CSC generation [113, 114]. Consequently, these results suggest that mitochondrial functions could be crucial in the survival of CSCs [109, 115].

There are several limitations which are beyond the scope of our review. Despite significant advancements in the field of cancer cell metabolism, more precise definitions and metabolic characterizations are required to avoid the misuse of the described terms in this chapter [99]. Based on these and the complexity of this subject, our recommendation is to focus on characterizing the metabolic characteristics and metabolic rewiring of stem/dormant and quiescent cells in a separate and comprehensive review.

## Metabolic plasticity and the main metabolic phenotypes in cancer

**Fig. 3:**
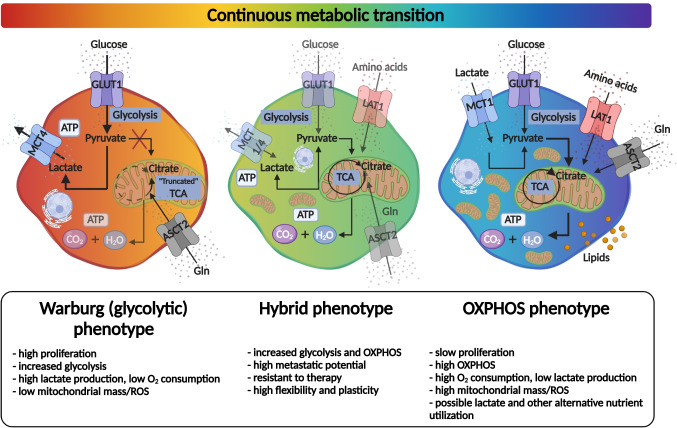
Metabolic phenotypes and their continuous transition in tumor tissues. Schematic presentation of Warburg (glycolytic), hybrid, and OXPHOS metabolic phenotypes. The metabolic rewiring (activated metabolic pathways are depicted in bold, in contrast to the less active routes which are faded). Additional phenotypic characteristics are labeled in the lower part of the figure

Evidence from ecology and biodiversity studies demonstrates the role of biological diversity in stabilizing the ecosystem (as part of a fluctuating environmental adaptation process). The presence of different, individual strategies is essential for some species’ survival because the absence of environmental compensatory mechanisms leads to their collapse. Diversity is necessary for maintaining stability and productivity of the cellular ecosystem [116]. The available literature in cancer research confirms that tumors generate highly diverse and unique ecosystems in the body, expanding the adaptation possibilities [3, 117]. High plasticity in extreme situations, such as nutrient, O_2_ starvation, and treatments with high cellular toxicity, could have significant advantages, especially at a cellular metabolism level. Metabolic plasticity, the interconnecting, flexible metabolic pathways, and rewiring cause a therapeutic nightmare during cancer progression. There are several attempts to define the various metabolic phenotypes of different cell types in cancer tissues. Based on database analyses, *Yu* *et al*. suggest that a minimum of three main metabolic phenotypes must be distinguished in tumor tissues. Aside from cancer cells with *glycolytic* and *OXPHOS* metabolic characteristics (two main types), the most dangerous cells could have the flexibility to utilize both OXPHOS and Warburg glycolysis simultaneously, and as a result, these cells have the highest metabolic plasticity [118] (Fig. 3). This newly defined *hybrid state* could have several advantages in a developing tumor, but targeting these hybrid cells, and other bioenergetic mechanisms, is not easy without causing potential side effects. Compared to normal cells (immune cells, fibroblast, adipocytes, etc.), the selective advantage of such a hybrid phenotype is the plasticity and rapid adaptation to utilize different bioenergetic sources (from the microenvironment or internal utilization of cells — autophagy) in cellular survival.

Apart from these three main metabolic phenotypes, there is a progressive and continuous transition in metabolic reprogramming, similar to epithelial–quasi-mesenchymal–mesenchymal transitions. Cells with different metabolic phenotypes and plasticity are constituents of metabolic symbiosis (promote tumor growth by supplementing each other). Moreover, metabolic heterogeneity could be the basis of tissue heterogeneity and tumor survival. The characterization of these findings and the discovery of potentially favorable master regulators as targets could improve the understanding and clinical outcomes (therapy resistance, relapse, and distant metastasis in advanced cancers) [39].

## Characterization of metabolic heterogeneity in cancer tissues

Next-generation sequencing is a relatively quick and sensitive technique for analyzing individual genetic variability; additionally, its routine application serves as a tool to identify alterations on genomic level and tailor precision and targeted therapies. Despite the early and, in some instances, more prolonged success with personalized precision oncotherapy (based on genotype mapping), the prognosis for many cancer patients remains poor due to disease relapse and development of resistance to therapy. The advancement and clinical benefits of molecular targeted therapy still have some limitations, and usually, the median progression-free survival (PFS) does not increase more than 6 months [119]. The available targeted therapies usually depend on cytostatic effects that reduce tumor growth and kill many malignant cells. However, a selective survival of highly aggressive tumor cells can occur for several reasons (e.g., the genome-based characterization may not take into consideration the adaptive landscape). Another critical aspect of cancer progression is that if most tumors are successfully eliminated by therapy, one or a few resistant phenotypes may remain. These surviving tumor cells exist in a less-competitive environment at the primary tumor site or distant metastasis (the so-called competitive release). Additional studies are required involving mapping tissue heterogeneity and identifying these potentially surviving cells for more precise targeting [120–122]. To discover the source of tissue relapse and evaluate its relevance in the clinical setting, better *in situ* characterizations by radiologists and pathologists are needed. Mapping metabolic heterogeneity could be an additional tool in this research field. Therefore, we need to integrate metabolic profiling and *in situ* metabolic characterization in precision oncology. Several factors must be considered: metabolic heterogeneity of the primary tumor tissue (at the time of diagnosis); potential metabolic adaptation mechanisms which are available during tumor growth and development; and treatment regimen inducing alterations, etc. Studying these aspects, potential new metabolic targets need to be identified to interact with the survival mechanisms in surviving and developing cancer tissue.

**Fig. 4:**
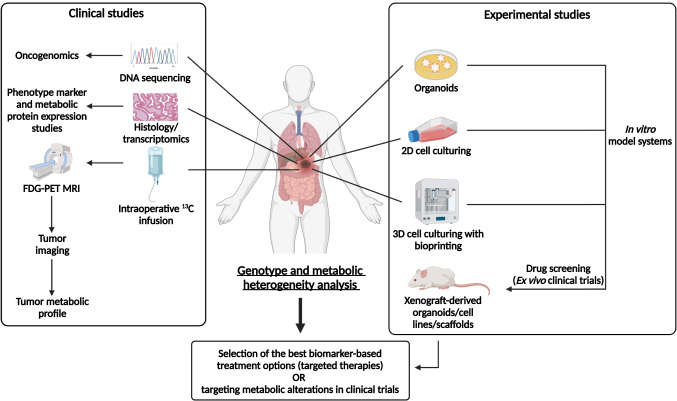
Metabolic characterization *in vivo*, in patients’ tumors, and experimental studies. Tumor diagnostic methods: clinical image analysis (PET-CT, MRI), tumor biopsy methods including histological and molecular genetic examinations (pathomorphological, immune, and next-generation sequencing–based phenotyping, and genetic analyses) are shown on the left side of the figure. Additionally, several *in vitro* and *in vivo* model systems and experimental studies using cell lines or patient-derived cells are shown on the right side of the figure

Intratumoral heterogeneity, including metabolic distributions and altering tissue microenvironmental conditions, must be mapped during the diagnostic process. Pathomorphological studies are necessary to apply this in practice with new mass spectrometry and imaging analyses in combination with *in vivo* imaging technologies. *In vivo* imaging methods, including the application of PET-CT and MRI [123], are potent tools for the following circumstances: (a) monitor *in vivo* metabolism, (b) diagnosis, and (c) follow the response rate of the therapy. This review could not summarize the available studies of these *in vivo* and *in situ* imaging technologies (MALDI imaging, CEST MRI, MRSI, IR-MALDESIMSI, SpaceM, single-cell spectrometry, Raman spectrometry, MetaSensor, etc. [124]). The overall advantage of these techniques is the ability to measure proliferation, necrosis, and inflammation by applying novel radiotracers and expanding evaluation methods. These methods must be complemented with the accurate pathologist-guided *in situ* metabolic characterizations of the available biopsies. Metabolic pathway–related expression and activity markers should be examined for *in situ* investigations of the tissue sections. To validate these markers, additional *in vivo* and *in vitro* metabolic profiling and studies are required. Furthermore, we need to synthesize DNA sequencing and transcriptomic data sets with the results of experimental metabolic studies (experimental metabolic profiling analyses of *in vitro* studies and patient-derived xenograft models) and *in vivo* metabolic imaging results (Fig. 4).

Metabolic pattern as a fingerprint is unique in each tumor tissue, and it depends on the tumor-specific cellular content (including non-tumorous cells: blood vessels, immune cells, etc.), genetic and epigenetic alterations, microenvironmental pressures (therapy, nutrient, oxygen stresses, etc.), and the metastatic sites in new organs. There were several attempts to characterize the metabolic activity and local differences in the central metabolic processes using immunohistochemistry (IHC) staining with different antibodies against metabolic enzymes, regulators, and transporters. Our primary interest is to map metabolic heterogeneity at tissue level and combine these data with the pathomorphological diagnosis.

In the last few years, the potential *hybrid metabolic phenotype* highlighted the interplay between glycolysis and OXPHOS. These results suggested that the high expression of HIF1α and p-AMPK could be the main features of hybrid cells. However, high HIF1α and low p-AMPK expression and their opposite distributions are characteristic for Warburg glycolytic and OXPHOS tumor cells, respectively [125, 126]. It was also proposed that hybrid metabolic cancer cells tend to progress highly aggressively and are associated with frequent metastasis and relapsed cancers [18]. These were examined using metabolomic and transcriptomic patient data sets diagnosed with breast cancer. However, some recent new results confirm that this hybrid metabolic phenotype could exist in both *in vitro* experimental models and tissue materials (both pathology and experimental studies) [118, 127, 128]. Further, especially pathological, studies are needed in these subjects. Recent studies focus on one aspect of the signaling regulatory failure–related metabolic alterations/characteristics, subtype-specific hyperactivation in specific metabolic processes, and nutrient utilization in different cancers. However, a comprehensive analysis of the whole network is not performed. Additionally, omics databases and integration algorithms can generate cancer-specific genotype, phenotype, and metabolic phenotypes of different cancers. Metabolic genes and their expression as cancer biomarkers can correlate with tissue acidification and migration or other cancer progression–related cellular functions [129, 130]. However, the selection, survival adaptation, and evolution of cancer metabolism at tissue level were suggested to optimize the growth potential of tumor cells in different cancers. The potentially helpful metabolic targets in extracellular and mitochondrial transporter mechanisms, peptide metabolism, FA synthesis, and PPP vary in different cancers [131–133]. The results of other pathomorphological and IHC combined biopsy studies are very diverse, and their conclusions depend on the applied scoring systems and whether tissue heterogeneity was investigated.

## *In situ* metabolic protein expression alterations and heterogeneity with respect to tumor progression

### Heterogeneity of carbon source and transmembrane trafficking

Tumor cell proliferation, survival, and migration are crucial elements in cancer progression. These processes have energy demands fueled by nutrients and metabolites sourced from additional bioenergetic mechanisms. The main metabolic pathways are connected to glycolysis and OXPHOS, preferentially using glucose as their energy source. Other properties that affect tumor survival and adaptation mechanisms include surrounding tissue microenvironment, biochemical factors, starvation, alternative nutrient utilization, and shifts in cellular metabolism. Healthy and nonmalignant cells usually produce pyruvate to fuel OXPHOS predominantly. During starvation, especially at low O_2_ concentrations, anaerobic glucose utilization is preferred, and alternative substrates are directed to fuel the TCA cycle. Tumor cells have metabolic plasticity and switch dynamically between lactate-producing Warburg glycolysis and OXPHOS. Tumor cells during significant proliferation use Warburg glycolysis preferentially due to *metabolic reprogramming* caused by several oncogenic mutations of the signaling network. The metabolic characteristics of cells are determined by the distribution of glucose, carboxylate, or amino acid transporters and the *carbon utilization* of the tissue. The expression patterns of specific transporters such as GLUT1, MCT1/MCT4, CAIX, and amino acid transporters (e.g., ASCT2 — alanine/serine/cysteine transporter 2, LAT1 — large neutral amino acid transporter 1) help characterize the supplementary nutrient processes within an *in situ* tumor mass. GLUT1 overexpression is characteristic for several cancers in correlation with their glucose utilization. This feature is useful to detect several relapsed and metastatic tumors with *in vivo* imaging diagnostic tools (e.g., FDG-PET-CT) depending on the tumor type. MCTs facilitate the lactic acid exchange between the cytoplasm and extracellular space. MCT1 is expressed in all cells and assists with the transportation of lactate and pyruvate. In contrast, MCT4 expression is inducible and responsible for the exportation of lactate under hypoxic conditions, e.g., in glycolytic tissues, especially in many cancer cells.

Experimental and imaging studies have extensively analyzed the expressions of *GLUT receptors*, *MCTs*, *carbonic anhydrases* (CAs), and other proteins (e.g., amino acid transporters). Accordingly, tumors are classified based on their levels of protein expression (high or low levels) [134]. However, the tissue distribution of transporters in various cancers is not yet well characterized. The available results focus on scoring total protein expression (staining intensity) using only high or low categories for GLUT1, MCT1/4, and CAIX. In certain studies, the staining level and pattern were described in correlation with hypoxia, HIF1α expression, and FDG-PET-CT images in various tumor types [135]. IHC documentations demonstrate intratumoral heterogeneity; however, this phenomenon was not evaluated/scored in pathology studies and Protein Atlas documentations.

A vast literature exists about oncogene-driven alterations and the prognostic role of GLUT1 expression. These findings suggest that, in general, difference in GLUT1 expression might be a significant predictor for overall survival (OS), disease-free survival (DFS), PFS, and disease-specific survival (DSS). Poor OS correlation is associated with several cancer types, e.g., in gastric, urinary, ovarian, oral squamous cell, pancreatic, colorectal, lung, gallbladder, and esophageal carcinoma [135]. Multi-tracer characterization studies with FDG-PET-CT have resulted in the ability to show a complete representation of glucose uptake, heterogeneity, and therapy-induced metabolic adaptation of cancers. CT- and MRI-driven imaging technologies can facilitate the mapping of metabolic phenotypes and guide metabolic treatments, e.g., lung cancer [136]. GLUT inhibitors have gained attention in correlation with the growing knowledge of the glucose dependency of cancer, inflammation, and other diseases [137]. Targeting GLUT receptors or glycolysis could be an alternative option in cancer treatment, but several clinical trials were discontinued because of the reported high toxicity and severe side effects (Table 1).

**Table 1:** Ongoing studies on glycolysis and metabolite transporter inhibitors

Target	Drug name	Indication	Highest development stage	Status
**Glycolysis inhibitors**				
Lactate dehydrogenase inhibitor	Nedosiran (DCR PHXC)	Primary hyperoxaluria	Phase III — NCT04042402	**Active**
Glyceraldehyde 3 phosphate dehydrogenase inhibitor	GP-2250	Pancreatic cancer	Phase II — NCT03854110	**Active**
	PS101 (3-bromopyruvate)	Solid tumor	Phase I — NCT04021277	**Active**
Hexokinase 2 inhibitor	Tuvatexib (VDA-1102)	Actinic keratosis	Phase II — NCT03538951	**Active**
Various targets	2-DG, lonidamine etc			**Inactive/discontinued**
**Transporter inhibitors**				
CAIX inhibitor	SLC-0111	Pancreatic cancer	Phase II — NCT03450018	**Active**
CD36 activator	Cyclopsaptide (VT-1021)	Solid tumor	Phase I — NCT03364400	**Active**
SLC7A5/LAT1 inhibitor	JPH-203	Bile duct cancer	Phase II — UMIN000034080	**Active**
Various targets	AZD3965, indisulam, MEDI7247			**Inactive/discontinued**

Overexpression of MCT1/4 is a poor prognostic factor for various cancers, including breast, bone, colon, and renal cancers [138]. MCT1 overexpression is associated with a worse prognosis in bladder, endometrial, and clear cell renal cell cancers [139–141] and with MCT4 in oral, colorectal, prostate, lung, and clear cell renal cell cancers (ccRCCs) [141–146], respectively. MCT4 expression is regulated by AMPK and protein kinase C (PKC), and these signals influence metabolic adaptations via lactate shuttle under specific conditions [147].

CAs — which have 16 different isoforms — are involved in the regulation of intra- and extracellular pH; their overexpression and different localization were described in various tumors. CAIX requires special attention based on its predicted diagnostic, prognostic, and therapeutic biomarker potential in solid tumor pathology [148]. Diffuse CAIX expression, similarly to high GLUT1 and HIF1α expression, is characteristic for ccRCCs (in contrast with papillary renal clear cell carcinoma — RCC). This can help in differential diagnosis; ccRCC has lower CAIX expression [149]. On the contrary, higher expression of CAIX is correlated with poorer prognosis in the majority of breast, lung, ovarian, oral squamous, liver, and bladder cancer cells and glioblastomas [150–156]. Considering this, CAIX could be therapeutically targeted [148]. In addition, IHC evaluation of MCT1/4 and CAIX — mainly focused on detecting higher or lower expression levels in tissues [66] — showed a correlation with hypoxia. Further research should be done to investigate tissue heterogeneity of CAIX and MCT4 expression, and its impact on the progression of solid tumors requires further analysis [157]. It was described that knockout (KO) or drug targeting of MCT1/4 inhibits the proliferation capacity both *in vitro* and *in vivo *[66]. Furthermore, these combined with metformin or phenformin [158] lead to synthetic lethality in tumor models [159]. Some effective small-molecule MCT1-specific inhibitors are in clinical trials (e.g., AZD3965), monoclonal antibodies, and small-molecule inhibitors of CAIX (SLC-0111) showing promising results. Some of these MCT1 inhibitors have entered phase II clinical trials, but the lack of isoform selectivity and potential associated toxicity necessitates designing additional, more selective drugs. Combining these with traditional chemotherapy or other metabolic targeting drugs during anticancer therapy is also being investigated [148, 149, 160] (Table 1).

Cellular uptake, transport, and the role of *amino acids* (as supplementary mechanisms) are well known in differentiated and proliferating cells. The primary function of amino acid metabolism is to serve protein synthesis. However, the complex metabolic coupling also supports the source of building blocks and other molecular precursors such as nucleotide, glutathione, and polyamine synthesis [161]. Furthermore, the emerging roles of non-proteinogenic amino acid metabolism in cancer should be considered. Nonessential amino acids limit tumor proliferation; in this case, the stromal and extracellular amino acids are often consumed and recycled. Amino acid restriction can be compensated by autophagy, and it could be mentioned that amino acid availability (intracellular concentrations) regulates the growth potential and influences stress responses, e.g., via mTORC1 activity [161–163]. Based on increased amino acid demand during intensive proliferation, some nonessential amino acids (e.g., glutamine, asparagine, arginine, and cysteine) become conditionally essential. Glutamine addiction, the importance of glutaminolysis (reversible glutamine-glutamate-αKG metabolic axis), and additional “by-products” provide building blocks for fast proliferating cells. It should be considered that glutamine can be nitrogen and carbon sources in amino acid, nucleic acid, and lipid synthesis. There are several glutamine exchangers (ASCTs and Na^+^-coupled neutral amino acid transporters (SNATs)) that are often overexpressed by tumor cells (e.g., ASCT2 — SLC1A5; SNAT1 — SLC38A1, SNAT2 — SLC38A, and SNAT5 — SLC38A5) [164]. There were promising results for inhibiting tumor growth with applying ASCT2 inhibitors in gastric, prostate, lung, and breast cancers [165, 166]. However, many compensatory mechanisms were detected (e.g., SNAT1 overexpression) negating the effect of these inhibitors [167].

Therefore, the generation of novel targeted therapies and new inhibitors is under development. Many other amino acid transport (symport, antiport) mechanisms have been reported and tested in the last few decades. Serine/glycine-linked metabolic network and the related transporters can be involved in different mechanisms in cancer progression. These transports are dysregulated via epigenetic alterations (e.g., methylation) or *de novo* ATP synthesis. In addition, the modifications in serine-glycine extracellular level could influence the proliferation and function of cells in the immune microenvironment (T cell expansion) [168]. The transport of leucine, isoleucine, and valine — essential branched-chain amino acids — can also be upregulated in common cancers (e.g., lung, breast, and prostate). They have a role in maintaining amino acid pools in tumors [169]. Recently, small-molecule inhibitors and their derivatives have been applied as actionable targets, e.g., LAT1 (SLC7A5). Additionally, LAT1 targeted inhibitors were tested *in vitro* and *in vivo*, where decreasing leucine level and inactivation of mTORC1 are correlated with reduced tumor growth. [170]. Some of these (e.g., JPH203) have recently been introduced to clinical trials in advanced solid malignancies [171]. The *de novo* biosynthesis of arginine occurs in the urea cycle during ammonia detoxification. Arginine is referred to as a conditionally essential amino acid that serves as a precursor for polyamines and generates NO, creatine, and other amino acids. Additionally, arginine can be taken up by cationic amino acid transporters. Overexpression of SLC7A1 cationic amino acid transporter and its role in tumor cell survival were described in arginine-dependent breast cancers and cases with multikinase inhibitor resistance. The characterization of amino acid transporter expression and their alterations have been described in different cancers at protein and tissue levels. Limited data are available about the heterogeneous staining of LAT1, ASCT2, or other transporters. Some recent studies have combined these with *in vivo* PET-CT images [172, 173]. This highlights that metabolic heterogeneity could be scored by *in vivo* analyses and by special pathomorphological studies of these transport mechanisms in the future.

More recent attention has focused on the provision of the *FA*, *acetate*, and *citrate* utilization processes which have renewed interest; FA and lipids contribute to new biological membrane synthesis during the intensive proliferation of doubling cells. FA can be served by exogenous uptake and *de novo* synthesis. During tumor development, lipid metabolism alterations occur such as FA transport, lipid storage (e.g., LD), *de novo* lipogenesis, and β-oxidation–mediated energy production [174]. FA translocases (FATs), CD36, FA transport proteins (SLC27 members — FATP1-6), and other FA-binding proteins in membranes (e.g., FABPpm) have been intensively studied. These transporters were described to be overexpressed in breast, gastric, pancreatic, hepatocellular, and prostate cancers [175–177]. It was also suggested that CD36 plays an essential role in metabolite exchange and symbiosis of the tissue microenvironment, facilitating lipid uptake of tumor cells [72, 178]. The occurrence of metastasis in visceral adipose tissue could be correlated with FA uptake and utilization. During progression, the adipose tissue generates an immunogenic inflammatory microenvironment and modulates paracrine-endocrine signals [179]. Lipid storage elevates LD formation, which dynamically contributes to lipid homeostasis by preventing lipid toxicity and providing acetyl-CoA, ATP, and NADH through β-oxidation [174]. Acetyl-CoA produced by β-oxidation from LDs assists the ATP generation in high amount via TCA cycle and electron transport chain. This process is highly effective since it gives a much higher amount of bioenergy than other mechanisms from carbohydrates. In parallel, the generated NADH is also helpful for ROS detoxification [180]. Besides lipid uptake, *de novo* lipid synthesis is characteristic for cancer tissues using LDs or other lipid sources [181]. The primary source is cytoplasmic acetyl-CoA from carbohydrates (through acetate and citrate) and amino acids (including glutamine), converted to FAs. Under metabolic stress (e.g., hypoxia and lipid deficit), cancer cells convert acetate and citrate using ACSS and ACLY. Additionally, acetyl-CoA carboxylases (ACCs) and FA synthase (FASN) facilitate palmitate generation in lipid synthesis.

In correlation with the importance of balanced lipid homeostasis, the complex pan-cancer analyses of the prognostic role of CD36 overexpression have been published recently and highlighted the tumor type–dependent increase in CD36 expression. Unfortunately, intratumoral heterogeneity was not evaluated in these studies; therefore, further analyses are necessary by pathologists in different tumor types and their metastases [182].

Many transport proteins and different channels are necessary to connect mitochondrial and cytosolic metabolism, providing the integrity, metabolic cargo, and maintenance of mitochondrial homeostasis. Most of the 53 mitochondrial carriers are localized in the inner mitochondrial membrane. *Mitochondrial carrier family* (SLC25) members are the most critical transporters; however, others such as pyruvate carriers and ABC transporters also have essential functions [183]. These amino acids, nucleotides/dinucleotides, carboxylates, ketoacids, and additional carriers use several exchange mechanisms. Pi carriers (PiC) are used for phosphorylation of ADP by ATP synthase. The overexpression of SLC25A1, mitochondrial citrate transporter, was described in primary lung cancers and their metastases, contributing to therapy resistance. SLC25A10 transports malate and succinate, which is linked to NADPH synthesis and the maintenance of redox homeostasis [184, 185]. For example, aspartate and glutamate mitochondrial carrier (SLC25A12) overexpression (via unlimited amino acid shuttle) supports aspartate utilization in hepatocellular carcinoma (HCC) [186]. Changes in mitochondrial metabolite transporter expression and their role in cancer progression correlated with ROS accumulation in central nerve system (CNS) neoplasms (overexpression of SLC25A30). On the contrary, SLC25A43 loss was detected in HER2 + breast cancers [187]. Based on these results, the alterations in mitochondrial carrier expression (see below), their potential impact on tumor growth, and inhibitor therapy are under investigation (e.g., SLC25A10 inhibitors, which disturb redox homeostasis).

Different metabolite transporters and their role in cancer progression are a current topic of interest. The expression patterns of the above-discussed transporters could expand therapeutic targets concerning the dynamic and complex adaptation mechanisms in cellular stress, altered extracellular nutrient concentration, and growing/survival demands.

### Tissue heterogeneity of certain metabolic enzymes and their significance in metabolic adaptation

There are many networks, data analysis, and IHC studies focusing on the changes in metabolic enzyme expressions, at tissue level. Most of these studies analyzed the changes (increase, decrease, or loss of some central metabolic enzymes) in various catabolic and anabolic processes (glycolysis, TCA enzymes, lipid synthesis or oxidation, amino acids, electron transport chain proteins, etc.). However, the description of staining heterogeneity in correlation with the clinical data are usually not evaluated.

In the past, the metabolic state of certain tumors was analyzed in granulomas of sarcoidosis, some rare tumors, and other diseases by applying traditional enzyme histochemistry to detect enzyme activity. For example, cyclooxygenase (COX), NADH, SDH, and ATPase activity could be shown by enzyme histochemistry on native slides; however, routine biopsy materials are mainly formalin-fixed. Nowadays, this staining has been replaced by specific IHC reactions in routine pathology. Instead of testing real metabolic enzyme activity in tissues *in situ*, the current approach evaluates the enzymes and their active forms.

Several studies describe the alterations of enzyme expression in correlation with hypoxia, increased HIF1α, and related altered tissue distribution of prolyl hydroxylases (PHDs) [188]. This highlights the role of the tissue microenvironment and its influence on metabolic alterations in various cancers and cancer progression. *In situ* HK2, LDHA, PKM2, PDKs, PDHs, factor inhibiting hypoxia (FIH), and different PHDs were studied in tissues over the last few decades. The upregulation of PKM2 expression, which is often associated with increased glycolytic enzymes or glucose transporters (e.g., HK2, GLUT), was described at tissue level in various cancers (e.g., in breast, lung, ovarian, bladder, colon cancers). The unfavorable prognosis (lower OS and DFS) was correlated with higher PKM2 expression, e.g., in breast, hepatocellular cancer, as well as in tongue and esophagus squamous cell carcinoma [189–192]. In correlation with glucose utilization, elevated HK2 was recently described in patients’ biopsies of esophageal, renal, lung, cervical, colon, and breast cancers [193–195]. The analyses of LDHA and PDK1 *in situ* protein expression also showed association with poor survival and therapy resistance in many malignancies (colon, breast, uterine, pancreas, lung, renal, gastric cancers, CNS malignancies, etc.). Considering the increasing serum lactate level of end-stage cachexia patients, these observations are not surprising [196–201].

Glutamine and glutamate utilization are essential supporters of TCA fulfilling mechanisms and redox homeostasis. GLS expression and tissue distribution were also intensively examined. These studies highlight that GLS expression is often increased during metabolic adaptation in malignant progression of prostate, breast, colon, ovarian, pancreatic cancers, gliomas, rhabdomyosarcomas, etc. These could be potential targets in rare malignant cancers (e.g., sarcoma, lymphangioleiomyomatosis, or pheochromocytoma) [202–207]. Other amino acids and their roles in metabolic network regulation are also crucial. Amino acid (e.g., leucine and arginine) sensing, transporters, carriers, and their cytoplasmic or lysosomal levels influence the intracellular anabolic and catabolic balance. This will be discussed below among mTOR activity regulation and its metabolic functions.

TCA cycle, OXPHOS, the mitochondrial electron transport chain enzymes, and their activity are also under investigation. However, their functional alterations are more common than changes in expression level. It was found that COX enzymes are overexpressed in many cancers; e.g., overexpression of COX2 is characteristic for breast, gastric, and prostate cancer; COX4/COX5B can be detected in glioma and renal and breast cancers. Additionally, increased COX2 level was described to correlate with worse prognosis in cholangiocarcinoma, gynecologic, and gastric cancers. However, there were contradictory COX expression results in other cancers (e.g., breast and cervical cancers) [208–212] regarding the differences in metabolic rewiring (tissue-specific). The ATP synthases level in various tumor tissues suggests that lowered ATP synthase expression and the reduced mitochondrial electron transport chain activity may have a predictive role in progression and prognosis (worse outcome in ccRCC, NSCLC, and colon cancer patients) [213–215]. An increased need for substrates places stress on the dynamic network, in which a back flux of protons can attenuate into the mitochondrial matrix (uncoupling). Mitochondrial uncoupling proteins (UCP1-5) have a role in nucleotide and FA cycling. As important mitochondrial transport mechanisms, these link mitochondrial respiration and ATP synthesis and decrease superoxide formation. UCP2-mediated metabolite antiport (aspartate, oxaloacetate, and malate) could cause special metabolism-rewiring in cancer cells which confer chemoresistance as well [216, 217]. Induction of UCP2 has been related to metabolic switch leading to accelerated glycolysis and reduced mitochondrial activity, e.g., in colon, pancreas, lung, breast, prostate, and head and neck cancers [218].

Lipids (triglycerides, phospholipids, sphingolipids, and cholesterol) can be used as an energy sources, building blocks of membrane components, and precursors for steroid hormone, bile acids, vitamins, etc. Based on these, cancer cells can completely rewire their lipid metabolism with increased *de novo* lipid synthesis, FA uptake, and FA oxidation and finally alter cancer-associated adipose tissues [174]. These central changes influence FA, cholesterol, arachidonic metabolism, and peroxisome proliferator–activated receptor (PPAR) signals. Elevated CD36 expression facilitates free FA uptake that supplies new membrane building and influences signaling events via activating, e.g., Wnt and TGFβ pathways [175, 176]. *De novo* lipid synthesis can be supported by acetyl-CoA production from glucose and glutamine via glycolysis or the truncated TCA cycle, respectively. Furthermore, hyperactivated FA synthesis is in correlation with an increased amount of crucial lipogenic enzymes (ACLY, ACC, CoA carboxylase — ACACA, FA synthase — FASN, and stearoyl-CoA — SCD). ACC and FASN are generally upregulated in growing tumors (breast, renal, gastric, colon, esophagus, lung, ovarian, prostate cancers, melanoma, gastrointestinal stromal tumors — GISTs, gliomas, etc.) [176, 219, 220]. Additionally, SREBPs activate the transcription of several enzymes participating in FA, cholesterol, and phospholipid synthesis in cancers (e.g., in NSCLC, pancreas, colon, endometrial, and breast cancers). The levels of many previously mentioned enzymes (ACLY, FASN, ACC) are increasing after SREBP1 activation propagated by PI3K/Akt/mTOR or Ras/Raf/Mek/Erk signaling pathways [221–224]. Furthermore, some studies indicate that a high level of SREBP1 protein expression was detected in specific tumor types by IHC, which could be an independent prognostic marker in breast, thyroid, and pancreatic cancers. Carnitine palmitoyltransferases (CPTs), localized in either the inner or outer mitochondrial membranes, and carnitine-acyl transferase (CAT) support the rate-limiting steps of FA β-oxidation via FAO, which can produce a high amount of ATP. Shifts among switching on or off, the oxidation of newly synthesized FAs, or their accumulation in LDs are regulated by extracellular tissue environment and local conditions. Moreover, starving conditions can provoke autophagy both in tumors and neighboring cells [225]. PPARγ participates in storing FAs as triglycerides in lipid droplets; therefore, this mechanism could rescue the cells from the toxic increase of endogenous palmitate [226]. LDH cholesterols are also essential components for membrane building in lipid rafts. Cholesterol homeostasis is balanced by *de novo* synthesis, uptake, and removal; the upregulated cholesterol synthesis is influenced by SREBP2 and orchestrated by oncogenic signals (PI3K/Akt, RAS/MAPK) [227]. The prognostic value of dysregulated FA acid oxidation, synthesis, lipid homeostasis, and expression alterations of contributing metabolic enzymes are known from several IHC studies. This highlights that lipid metabolism–related enzyme expression changes could be targeted (e.g., ACC, CPT1A, ACLY, or FASN) since these alterations could correlate with patients’ prognosis [128, 202, 205, 228–231]. Recent *in situ* human tumor tissue studies have investigated only some elements of these networks; therefore, its complexity is not well examined, and a deeper characterization is necessary.

In case of lipid metabolism regulation, the role of altered oncogene-addicted metabolic rewiring was described in many details. The consequences of activation and frequent mutations of *RAS* and other cellular signaling network elements (e.g., growth factor receptor kinases, TSC1/2, PTEN, PI3KCA in colon and breast cancers, and NSCLCs) contribute to the Erk1/2 and mTORC1/C2 hyperactivity by many steps (regulation of mitochondrial biosynthesis and lipogenesis, SREBP1 maturation) [232, 233]. Additionally, mTOR phosphorylates and inactivates lipin1 to liberate SREBP1. It is well-known that mTOR inhibitor therapies or Raptor KO decrease the expression of lipogenic enzymes FASN, ACLY, and ACC [234, 235]. Moreover, mTORC1 activation has translational effects by activating ribosomal S6K and ribosome genesis and influencing spliceosome formation [236]. The recent publications and ongoing studies suggest that mTORC2 can also affect lipogenesis. mTORC2 phosphorylates PKC isoforms, SGK1, and ACLY, therefore increasing activity of ChREBP, histone acetylation, and lipogenic gene expression in adipocytes [237]. These underline the specific regulatory role of mTOR signal activity in lipid metabolism.

Metastasis-driven cellular alterations (EMT-invasion-intravasation-extravasation-MET-metastatic growing) are cooperating in a complex manner during metastatic progression. Various molecular mechanisms influence these programs; these related cellular processes reprogram metabolic signals and regulate the expression of related enzymes. Additionally, metabolites produced by cancer cells/tissues stimulate migration, survival of circulating cells, colonization, and regrowth of tumor cells in a specific new microenvironment. Several experimental and clinical data underline the role of glycolysis (Warburg glycolytic process) in metastatic progression. In animal models, c-Src tyrosine kinase–mediated HK1/2 increase was documented in lung metastasis of colon cancers [238]. In other experiments, the silencing of HK2 reduced lung metastasis in several cancer models [239].

Moreover, the accumulation of glycolytic methylglyoxal and the glycated proteins activate EMT and migration in breast and colon cancer metastasis [240]. Increased amount and nuclear localization of PKM2 are correlated with the metastatic properties and behavior of many epithelial cancers [241]. Glycolytic lactate has additional critical regulatory functions in metastasis formation and progression (e.g., β-integrin, MCT expression alterations, or increasing MMPs). As a part of glucose usage, PPP is a primary source of NADPH that can neutralize ROS in detached and regrowing cells. In contrast, cells with lost anchorage have reduced proliferation, glucose uptake, and PPP pathway activity, increasing the ROS level. In this context, it was described that metabolic rewiring could be microenvironment-dependent in breast cancer metastasis; G6PDH and glutathione reductase levels can be increased in brain metastasis. However, in bone metastasis, the opposite is observed [242]. It was published that PPP can be upregulated in metastatic versus primary tumors in melanoma, renal, and pancreatic cancers [243]. Oxidation of glutamine, FAs, and glucose in TCA is also linked to cancer cell progression and metastasis formation [244, 245]. Glutamine and FA oxidation were described as characteristic features of specific metastatic cancer cells, e.g., melanoma, prostate, and breast cancer [246–249].

**Fig. 5:**
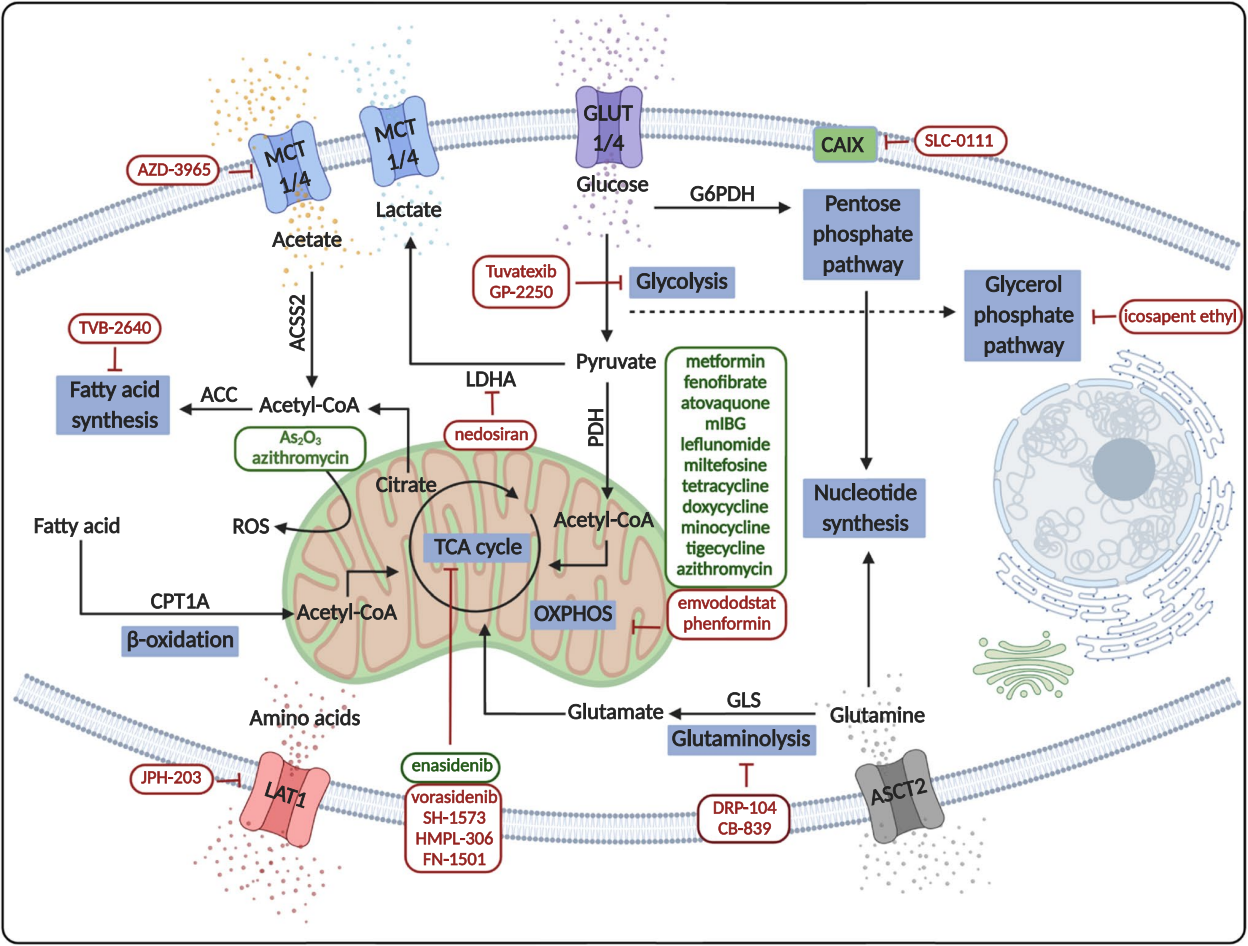
A simplified summary of the main targetable metabolic pathways and related compounds. The schematic figure shows the main metabolic pathways and inhibitor therapy reported in different phase trials (marketed and tested drugs are labeled with green or red color, respectively). OXPHOS inhibitors, metformin (marketed in type 2 diabetes), fenofibrate (marketed in different cardiovascular diseases), atovaquone (marketed in malaria), arsenic trioxide (marketed in acute promyelocytic leukemia), mIBG (marketed in paraganglioma and pheochromocytoma), enasidenib mesylate (marketed in relapsed acute myeloid leukemia), leflunomide (marketed in psoriatic and rheumatoid arthritis), miltefosine (marketed in leishmaniasis), tetracycline, doxycycline, minocycline, tigecycline, azithromycin (marketed in bacterial infections), pyrvinium pamoate (FDA-approved anthelmintic drug) and drugs in clinical trials are vorasidenib (NCT04603001 — phase III in advanced hematologic malignancies), emvododstat (NCT03761069 — phase I in relapsed acute leukemias), FN-1501 (NCT03690154 — phase I in advanced solid tumors and leukemias), HMPL-306 (NCT04764474 — phase I in IDH gene mutated cancers), and SH-1573 — phase I in acute myelogenous leukemia); glycolysis inhibitors, nedosiran (NCT04042402 — phase III in primary hyperoxaluria), GP-2250 (NCT03854110 — phase II in pancreatic cancer), tuvatexib (NCT03538951 — phase II in actinic keratosis), PS101/3BP (NCT04021277 — phase I in hepatic metastases); lipid metabolism inhibitors, icosapent ethyl (marketed in different cardiovascular diseases), TVB-2640 (NCT03179904 — phase II in advanced breast cancers); glutaminolysis inhibitors, DRP-104 (NCT04471415 — phase II in advanced solid tumors), CB-839 (NCT03047993 — phase II in advanced myelodysplastic syndrome); transporter inhibitors, AZD-3965 (progressing to phase II trials), SLC-0111 (NCT03450018 — phase II in pancreatic ductal cancers), JPH-203 (pre-registration in bile-duct cancer)

PGC1a could have tumor type–dependent effect in mitochondrial oxidation processes: PGC1a was described as a metastatic promoter expressed in circulating breast cancer cells [250]; however, its KO could increase the invasiveness of melanoma cells through focal adhesion kinase signaling, modulating cell–cell, cell–matrix connection, and support dissemination [251]. Even in starving conditions, the low amount of ATP (bioenergy) liberated from nutrients can serve migration and invasion, protect the cells from apoptosis and immune system attacks, and promote survival in a new organ. In correlation with these, some TCA intermediates could alter epigenetic regulation. For example, it was suggested that increased αKG could play an essential role in maintaining stemness [252]. Regarding this, ketoglutarate dehydrogenase inhibition can reduce metastasis in various models [253]. These examples underline that glycolytic and mitochondrial alterations can have anti- or pro-metastatic effects, depending on the tumor type and cellular microenvironment. These strongly emphasize the role of altered metabolism during metastasis [254]. Defining the metabolic profile in various cellular states (during the metastatic cascade) could expand therapeutic targets and precision-based therapy. Based on this growing evidence, metabolic enzyme inhibitors have been tested in many preclinical and clinical studies. These ongoing studies focus on conventional treatments where metabolic adaptation could have a role in developing resistance (Fig. 5). Based on the results from these trials, therapeutic options can be successfully tailored to each patient individually using metabolic inhibitors in combination with conventional therapies. However, undesirable side effects from combined treatment require special care by clinicians.

## mTOR as a master regulator in metabolic adaptation and mTOR hyperactivity in cancer

Examining metabolic symbiosis *in situ* is and correlating this data with patient survival and therapy response can be challenging. mTOR has been considered a master regulator in metabolic adaptation, and mTOR hyperactivity is characteristic for the majority of cancers. Over the last decades, there have been several attempts to characterize the main drivers of metabolic rewiring and their impact on cancer progression. Recently published studies are limited, and tissue heterogeneity was less studied in these works. Many studies have focused on some elements of PI3K/Akt/mTOR pathway. According to these results, it was suggested that this axis could have a special role in regulating metabolic symbiosis, maintaining bioenergetics, and providing building blocks for tumor growth at the primary and metastatic sites. Since mTOR kinase is at the central crossroad of the cellular signaling network, it has many metabolic sensory and effector functions. Accordingly, it is considered the master checkpoint in metabolic adaptation.

Almost 50 years ago, a small molecule was purified from *Streptomyces hygroscopicus* after an expedition to Easter Island (*Rapa Nui*) [255]. The discovered “rapamycin” gained more interest in the 1980s when its immunosuppressive effects were beginning to be investigated and applied in kidney transplantation [256]. The cellular binding partners and targets of this compound were identified in the 1990s by studying the rapamycin-resistant *Saccharomyces cerevisiae* [257]. Additionally, the mammalian/*mechanistic target of rapamycin (mTOR)* kinase and cellular targets were also isolated after *M.N. Hall’s* discovery. Their functions were characterized with the help of several scientists, and it was demonstrated that mTOR kinase exists in two different protein complexes, which have other cellular processes and inhibitor sensitivity [233, 258–264].

Emerging data provide additional links of mTOR complexes to many major signaling pathways and physiological functions maintaining cellular homeostasis. These confirm the central role of mTOR in regulating cellular survival, growth, and differentiation in the continuously changing surrounding microenvironment.

In parallel, it was described that rapamycin or rapalog (derivatives of rapamycin) treatment reduce tumor growth in renal cancers developing in kidney transplanted patients. Later, it was observed that the incidence of posttransplant malignancy decreased [265] in rapalog converted immunosuppression. Identifying the contribution of mTOR activity in cyclin D1 overexpression in mantle cell lymphomas highlighted the possible use of mTOR inhibitors (mTORIs) in lymphomas and other malignancies [266]. To date, mTORIs have been introduced into the therapy of several cancers: renal cancers, mantle cell lymphomas, advanced breast cancers (hormone receptor-positive), pancreatic, gastrointestinal, pulmonary neuroendocrine cancers, and sporadic lymphangiomatosis. The application and development of mTORIs are ongoing; currently, oncologists prescribe them as a personalized therapy regimen or combined therapies.

### mTOR complexes and their sensing functions in cellular fitness and growth potential

The two functionally and structurally different mTOR complexes (mTORC1 and mTORC2) consist of two common proteins, mTOR serine/threonine kinase, and mLST8. The specific subunits are composed of unique scaffold proteins, Raptor in mTORC1 and Rictor in mTORC2, respectively. The mTORC2 complex has additional elements such as mSIN1, MAPK-interacting protein 1, which determine the subcellular localization of the complex and help in substrate recruitment. Additionally, the endogenous common inhibitory protein (DEPTOR) can bind and inhibit mTOR kinase in both complexes, while PROTOR1/2 can associate with Rictor and inhibit mTORC2 specifically [267]. Crystallographic analysis revealed that dimerized protein complexes have different structures in different cellular localizations (membrane, lysosomes, endoplasmic reticulum — ER, etc.). These offer binding sites for several other regulators (Rheb GTPase), substrates, and protein complexes [268]. mTOR complexes integrate different external and internal information (signals) based on monitoring cellular stress conditions; these influence “cellular decisions” (proliferation/differentiation or growth/survival). These decisions are assisted by mTOR-related signaling crosstalk, networking with many pathways, cellular state sensing, feedback, and feed-forward regulatory mechanisms [269].

The environment and the actual condition of cells influence the cellular programs and functions. mTORC1 and mTORC2 complexes integrate several signals, including growth factors, nutrients, starving conditions, energy, and stress levels, to regulate their activity. mTORC1 should “turn on and off” in correlation with cellular bioenergetics, genome integrity status, energy, O_2_, and growth factor levels [264]. To monitor bioenergetics and the activity of growth signals, two small G protein families, the Rheb and Rag GTPases, alter mTOR kinase phosphorylation status, activity, and the cytoplasmic/lysosomal localization of mTORC1 complex [270]. Signals of cytokines and endocrine hormones maintain *Rheb in active GTP-bounded forms and determine the lysosomal localization of Rheb.* The translocation of the mTORC1 complex to lysosomes mainly depends on the presence of amino acids and glucose. Their appropriate concentrations activate *Rag GTPases and induce lysosomal localization of the mTORC1* complex [271]. Adequate levels of lysosomal cholesterol and arginine influence the lysosomal sensor (SLC38A9) and generate the activity of Rag GTPases [272]. GATOR1 usually shifts to mTORC1-Rag-GDPs; therefore, inhibited GATOR1 serves the RagA/B-GTP isoform and localization of mTORC1 to the lysosome [273, 274]. Additionally, GATOR1 functions can be inhibited by the folliculin sensor (high total amino acids level) and GATOR2 amino acid sensors (high level of arginine and leucine). Low levels of leucine, arginine, and their metabolites can activate Sestrin1/2/3, SAMTOR (S-adenosyl methionine regulator), and SARB1, leading to the inhibition of GATOR2. Therefore, active GATOR1 shifts RagC/D to GDP-bound forms, and mTORC1 loses the lysosomal membrane localization [275–277]. Moreover, lowering amino acids through Sestrin1/3 can alter the expressions of uncoupling proteins which switch off mTORC1 activity amino acid sensor pathways, independently [278]. This nutrient and growth factor sensor regulation provides the balance between anabolism and catabolism depending on nutrient conditions. Additionally, the *mTOR activating function of lysosomal Rheb GTPases is negatively regulated by TSC1/2.* The inhibitory effect of TSC1/2 can be maintained (reactivated) by hypoxia, energetic stress (e.g., low glucose and ATP level) — AMPK activation, DNA damage, or Wnt signaling. However, it can be suspended by several growth factors (e.g., IGFR, EGFR) [279–281].

**Fig. 6:**
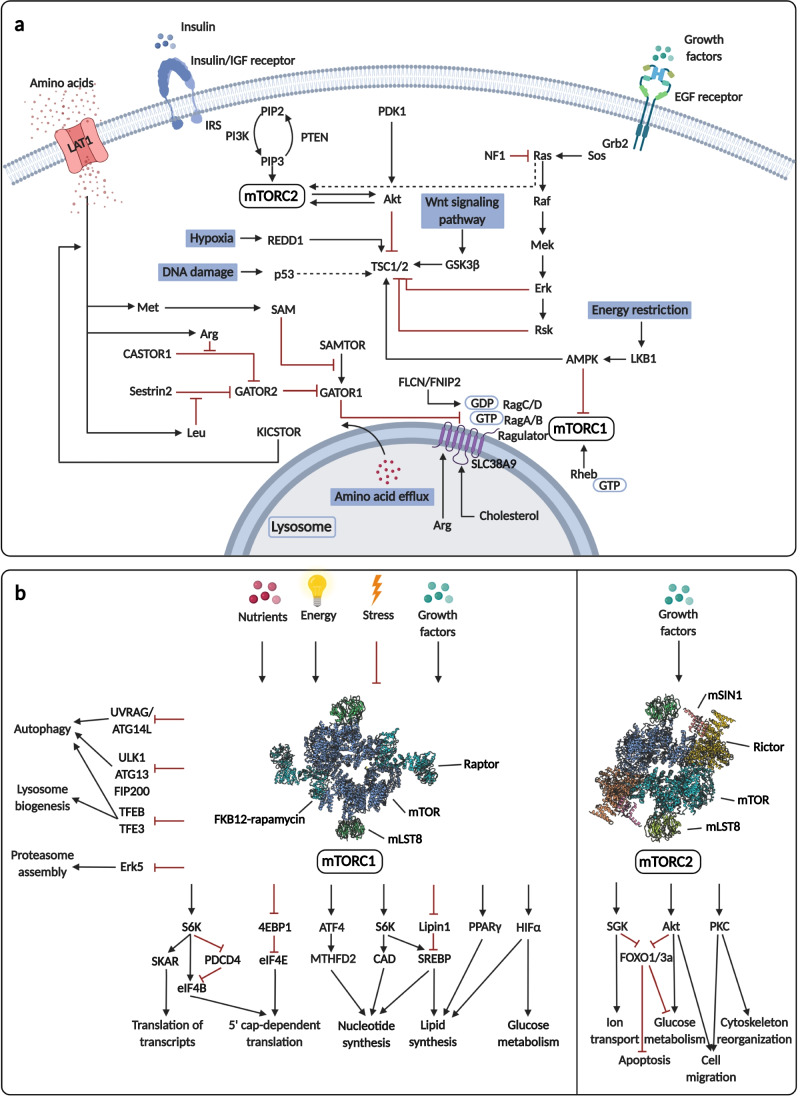
mTOR as a metabolic crossroad in the signaling network (simplified figures). **a** mTOR kinase and the mTORC1 and C2 complexes are metabolic and “cell-state” sensors in the signaling network. **b** The effector mechanisms and targets of mTORC1 and C2 complexes and their cellular regulatory functions. The activating (black) and inhibiting (red) effects are shown in the schematic diagram.

To date, the regulation of mTORC2 complex activity has been less described. Usually, the mTORC2 complex is positively regulated by growth factors mediated by PI3K pathway [282]. *mSIN1 pleckstrin domain autoinhibits mTORC2 activity*; this can be released by insulin- or serum-induced PI3K activation and increased PIP3. After these alterations, PIP3, mTORC2, and Akt are recruited into the cell membrane. mTORC2 is mainly active in the plasma membrane, but its active forms were also detected in the outer mitochondrial membrane and endosomes [283]. Evidence showed that growth factors regulate mTORC2; however, amino acids and other metabolites could also have some regulatory effects. Several new data highlighted that Rac1, Rap1, and Ras GTPases had potential roles in mTORC1 and mTORC2 activation and membrane localization processes in the last few years. These, especially oncogenic Ras, can directly induce mTORC2 kinase activation, promoting proliferation, and cell survival, which are associated with poor patient outcomes [284]. It was reported that lowered glutamine concentration can activate mTORC2 and modulate glutamine homeostasis by increasing glutamine-fructose 6 phosphate amidotransferase 1 and cellular catabolism [285]. mTORC2 complex activity can also be regulated through Rictor phosphorylation and acetylation. For example, GSK3 phosphorylates Rictor, initiating the FBXW7 ubiquitin–proteasome degradation of Rictor. It was also described that Rictor acetylation increases mTORC2 activity. Additionally, glucose stress and starvation activate AMPK, increasing mTORC2 activity in cells (instead of inhibiting mTORC1). These mechanisms underline the importance of mTORC2 functions in cell survival mechanisms under the conditions of starvation and acute bioenergetic stress (Fig. 6).

### Cellular fitness regulated by mTOR activity, controls proliferation, growth, and metabolic rewiring

“mTOR controls almost everything” — this statement highlights that mTOR plays crucial roles as it not only promotes cellular growth by stimulating proteins and initiates the synthesis of building blocks (from various sources: nucleotides and lipids) but inhibits autophagy as well.

The switch from anabolism to catabolism and vice versa is primarily regulated by mTORC1 activity. For example, in *in vitro* cell cultures, the growing cells are bathed in nutrients (growth factors, glucose, and proteins). In correlation with this, tumor cell lines have higher mTOR activity than their original *in vivo* tumor tissue counterparts (xenograft or human cancers *in situ*). However, in *in vivo* tumors, the fluctuation between fasting and feeding and the deregulated mTOR cause metabolic alterations under various environmental conditions [286, 287]. Several data confirm the importance of mTORC1-dependent metabolic regulatory functions in the whole organ and organism as well. After a 24-h fasting period in mouse models, it was observed that liver cell mass was reduced by 25% mTORC1 dependently (in case of Raptor KO mice, this reduction did not occur) [288]. Additional experiments described that mTORC1 activity delays autophagy under perinatal fasting conditions. The disturbance of these pathways results in uncompensated glucose reduction and mortality in animal models [289]. Moreover, hundreds of results underline the central role of mTORC1 in dysregulated metabolic signaling during overfeeding, obesity, and type II diabetes. It is not surprising that mTOR inhibitory treatments could decrease symptoms of obesity and diabetes [233]. mTORC1 activity is a well-known promoter of cellular protein, nucleotide, and lipid synthesis. These are in correlation with the already described effector mechanism of mTORC1: (a) phosphorylates eukaryotic initiation factor 4E–binding proteins (4EBP1) releasing eIF4E and enhances cap-dependent mRNA translation; (b) phosphorylates p70 S6 kinase 1 (S6K1) and subsequently ribosomal S6, which enhances the translational efficiency by influencing the expression and function of several ribosomal proteins (SKAR, RNA polymerases) and protein synthesis [290–292]; (c) phosphorylates and inactivates lipin1 and induces the translocation of SREBP1 from ER to the nucleus in an S6K1-dependent manner, initiating the expression of enzymes in lipid and cholesterol syntheses [293]; (d) phosphorylates ATF4 (activating transcription factor 4) and consequently induces MTHFD2 (mitochondrial tetrahydrofolate dehydrogenase) enhancing purine biosynthesis [294]; (e) phosphorylates and activates carbamoyl phosphate synthase (CAD), the rate-limiting enzyme in pyrimidine synthesis [295]; (f) influences several further transcription factors regulating hypoxia, energetic stress responses, or lysosomal biogenesis (HIF1α, PGC1a, TFEB) [296]. In parallel, mTORC1 activity decreases the catabolism in tumor cells by inhibiting the phosphorylation of autophagosome proteins ULK1 kinase and Atg13 autophagosome compartments, arresting autophagosome functions, and inducing autophagosome accumulation. Furthermore, it inhibits autophagosome maturation through phosphorylating other proteins by reducing lysosome biogenesis (UVRAG, Rab7, TFEB, TF3) [297, 298].

mTORC2 complex has been less characterized. However, its effector mechanisms were described in correlation with kinase activity, phosphorylation of AGC kinases, Akt, PKC members, and SGK1. The best-characterized kinase activity of mTORC2 is Ser473-Akt phosphorylation which is complex and context-dependent. The Ser473-Akt phosphorylation is required to phosphorylate some additional Akt substrates, including PRAS40, FOXO1/3a, and TSC2. It was suggested that this Ser473 phosphorylation is more critical for maximal activity of Akt than substrate specificity. Other phospho-Akts (Ser477, Thr479) resulting from mTORC2 induce the degradation of IRS1 by stabilizing FBW8 ubiquitin ligase activity [299]. mTORC2 is also stated to promote ACLY phosphorylation which increases ChREBP activity and influences histone acetylation [237]. mTORC2-mediated Akt phosphorylation could further promote c-Myc expression and, in turn, increase glycolytic HK2 expression and glucose-6-phosphate level. PKC phosphorylation by mTORC2 was suggested to regulate actin polymerization, which could orchestrate EMT, migration, and metastatic events [300]. Moreover, TGFβ, Wnt, and YAP/Hippo can also activate mTORC2, which could influence EMT-associated migration and invasion [282]. In a mouse model study, the role of mTORC2 in lipid metabolism was also highlighted. This detailed analysis confirmed mTORC2-mediated promotion of FA and lipid metabolism (sphingolipid and cardiolipin biosyntheses), causing steatosis and tumor development in the liver. Regarding these results, elevated mTORC2 and correlated lipogenesis were detected in human HCC cases. Besides the well-known regulatory functions of mTORC1 in autophagy, mTORC2 activity influences SGK-1– and Akt-mediated alterations of autophagy-related proteins (Atg7, beclin-1, FOXO3, VDAC1, etc.). In correlation with this autophagy regulating function the inactivation of mTORC2 and lowered SGK-1 activity, disrupting the autophagy and the normal differentiation in an animal model (*Caenorhabditis elegans*) [301, 302] (Fig. 6).

mTOR hyperactivity has multifunctional tumor growth–promoting effects. High mTOR activity (both complexes) through a broad spectrum of mechanisms contributes to cellular adaptation. mTOR hyperactivity has a central regulatory role in integrating complex pathway networks, especially in adaptation to bioenergetic demands and survival of migrating cells which can metastasize and be reactivated at distant sites. Therefore, it is not surprising that mTORC1 and mTORC2 complex overexpression and hyperactivity were described in many cancers, especially in patients with unfavorable prognoses [303, 304].

### Characterization of mTOR hyperactivity and its correlation with cancer progression and metastasis

Higher mTOR activity scores are associated with a worse prognosis in several tumor types (metastasis, leading to metastasis-related cancer mortality). During cancer progression, malignant cells lose their adherence capacity, gain invasive nature, survive under extreme conditions (e.g., in circulation), spread, and find new sites to form additional tumors. Tumor cells need to adapt to the new microenvironment and rearrange their resources, dependencies, and cellular connections during all these steps. Therefore, the mTOR activity–provided EMT-MET could contribute to the migratory and invasive potential of tumor cells in the metastatic cascade. Several publications confirmed that small GTPases support actin-cytoskeletal rearrangement, migration, and invasion. The contribution of mTOR signaling elements was described in correlation with TGFβ-mediated protein expression changes of EMT. Based on this observation, rapamycin can inhibit the TGFβ-dependent PI3K/Akt/mTOR activation and influence the migratory and invasive features of cancer cells. It was also described that mTORC2 complex–mediated functions are required in EMT, migration, and metastasis [305]. In prostate cancer, Rho and Rac1 GTPases were proposed to be regulated by mTOR [306]. Additionally, dual mTOR inhibitors were shown to arrest hypoxia- and TGFβ-mediated EMT in cancer cell lines. Besides these effects, mTOR activity–dependent alterations can induce protein expression (e.g., that of BMP2 and Sestrin2) and promote EMT and metastasis in different cancer models (ovarian, nasopharyngeal, pancreatic cancer, NSCLC, and HCC) [307]. Both mTORC1 and mTORC2 could contribute to modifying the metastatic properties of cells. It was described that IGF induces mTOR/Raptor/S6K1–dependent F-actin reorganization and phosphorylation of focal adhesion protein [308]. It was also published that the knockdown of mTOR/Rictor/mLST8 resulted in malformation of F-actin fibers and prevented paxillin phosphorylation, leading to the recruitment of focal adhesion complexes in starved fibroblasts [309–311]. The dynamics of cytoskeleton reorganization were found to be mediated by mTOR, mTORC2 via the Rho family of small GTPases (Rho A, Cdc42, and Rac1) in RH30 and HeLa cells. It was also proposed that mTORC2 controls the migration of neutrophils via Rac/Cdc42 [312, 313]. Recently, it was confirmed that mTOR KO caused dis-morphogenesis (documented by morphology analyses) in correlation with actin synthesis and assembly (F-actin and Cdc42 expression) in tooth epithelia [313].

Genetically modified Rictor expression influences the metastasis formation of HER2 overexpressing mouse breast cancers. The studied metastasis model system documented that mTORC2 can activate Rac1 into two ways, either via decreasing the expression of RhoGDI2 inhibitor by PKCa or via Akt-activated Rac-GEF Tiam1 [314]. Additionally, data obtained from mTOR/Rictor/PKCa silencing studies confirmed the role of mTORC2 in PKCa-mediated actin filament organization [315]. Following these results, mTOR inhibitor therapy decreases cell motility and metastatic potential in several preclinical and clinical studies (OS and DFS studies were performed).

**Fig. 7:**
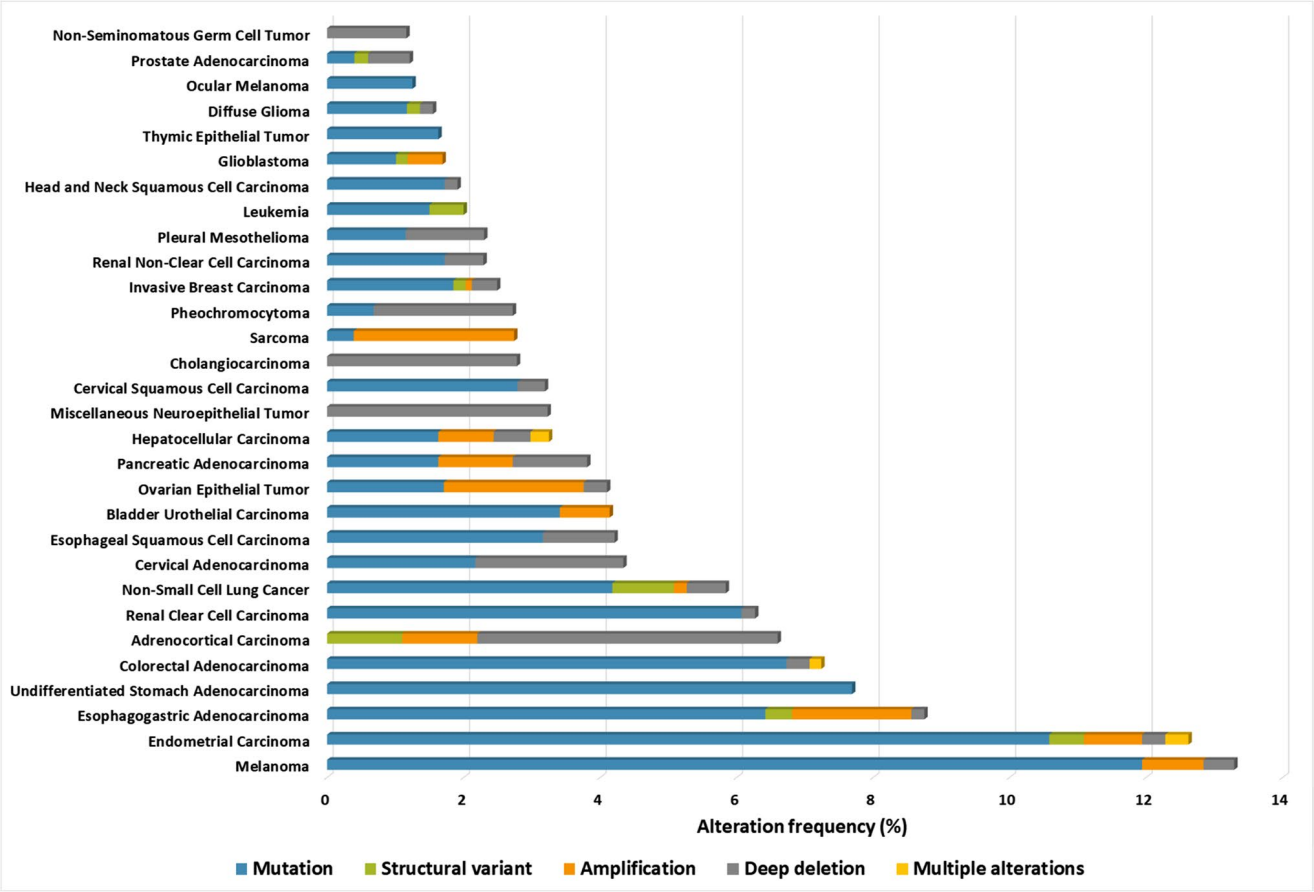
Genetic alterations of *MTOR* gene and their frequency in human cancer (point mutations, structural alterations — fusions, amplifications, deletions, or multiple alterations) are shown based on TCGA databases

Receptor tyrosine kinases (EGFR, HER2, PDGFRa, and FGFR in colorectal, breast, head and neck, gastric cancer, NSCLC, glioblastomas, GISTs, melanoma, etc.), PI3KCA and *RAS mutation-mediated* PI3K hyperactivity, as well as the loss of *PTEN* or *TSC1/2*, leading to mTOR hyperactivity in many cancers. Additionally, amplification and mutations of mTOR kinase and Rictor could also influence mTOR deregulation in cancers. *PI3KCA*, *AKT1*, *PTEN*, *TSC1/2*, or *LKB1* modifications and *PI3KCA* alterations are frequently described in breast (> 20%), colon, and gynecologic cancers. *PTEN* mutations occur in colorectal cancers and about 10% of central nervous system malignancies. *TSC1/2* loss can be detected in 4–6% of endometrial, urothelial, cervical, liver, and lung cancers. On the contrary, loss-of-function mutations occur less frequently in the prostate, endometrium, breast cancers, melanomas, glioblastomas, and renal cancers (in less aggressive hamartomas, rhabdomyoma, and angiofibroma). The deregulation of these growth signaling pathways correlates with mTOR hyperactivity. *mTOR* mutations and *RICTOR* amplification are among the most significant and frequent oncogenic mutations. Approximately, 5% of solid cancers carry disease-related mTOR kinase activating mutations (e.g., in melanomas, colon, renal, lung, endometrial, and gastric cancers) (Fig. 7). The importance of *RICTOR* amplification and overexpression was described in small cell lung (SCLC), breast, gastric, and head and neck cancers [316–319]. As a result of cellular signaling network–related oncogenic alterations, increased mTOR activity can be detected in ~ 80% of human malignancies. Consequently, mTOR hyperactivity and its outcome on a cellular level can influence the therapeutic sensitivity and progression (in a tumor type–dependent manner). mTOR hyperactivity can overwrite the metabolic checkpoints, contributing to cancer growth nutrient and growth factors independently.

Studying the mTOR hyperactivity of malignancies at the protein level was initiated in 1998. Studies examined p-mTOR and p-p70S6K proteins in malignant cell lines were initially conducted by Western blot analyses [320]. The development of phospho-protein antibodies and further examination of mTORC1 and mTORC2 targets could be possible with *in situ* characterization of human biopsy materials (formalin-fixed tissues).

Experimental data found that the pattern of mTOR hyperactivity could be altered at the tissue level during therapy [321]. Additionally, the progression and metastatic potential and the preference of metastatic sites were analyzed in correlation with mTOR hyperactivity in different cancers [322]. However, higher mTOR activity was described in many various cancers; the *in situ* staining pattern (heterogeneity) and the mTORC1/C2 complex distribution were not studied in detail. More precise analyses of tissue and mTORC1/C2 complex–related mTOR activity distribution, special markers, the use of well-defined complex scoring systems, and excellent pathologists are required. Moreover, active (phosphorylated) forms of mTOR kinase, other mTORC1, and mTORC2-specific elements (e.g., Raptor and Rictor) and active forms of mTOR complex target proteins (p-S6K1, p-4EBP1, pS6, p-Ser473-Akt, p-SGK1, etc.) must be analyzed.

Many studies on mTOR hyperactivity and its correlation with prognosis have been published; however, mTORC1 and mTORC2 complex distribution and additional metabolic regulatory proteins and their correlation with tumor progression have been less studied [323]. In these studies, strict conclusions were not possible due to lower case numbers, a broad diversity of tumor subtypes (with higher incidence), and intertumoral differences. In the early 2010s, our and others’ results confirmed that elevated mTOR activity is characteristic for human lymphoma and leukemia cells. Based on IHC and flow cytometry data, higher mTOR activity, especially in the presence of a high amount of mTORC2 complex, was correlated with worse prognosis and lower OS in lymphoma/leukemia patients [324–327].

In the case of solid tumors, similar observations were published in the last decade. For example, almost all of the published studies underline the role of mTOR hyperactivity in the progression and metastasis formation in hormone receptor–positive (HR +) or triple-negative (TN) breast cancers. Recent preclinical studies described that mTORC1 and mTORC2 inhibition could have significant growth inhibitory effects in these malignancies [328–330]. Moreover, in some reports, it was also highlighted that high mTOR activity could be associated with the presence of metabolic plasticity (higher expression level of alternative metabolic enzymes at tissue level, e.g., simultaneously increased expressions of LDHA, GLS, and carnitine palmitoyltransferase 1A — CPT1A) in breast and other cancers [128, 202, 331–333]. Our findings suggest that metabolic plasticity (high mTOR activity and the overexpression of at least two alternative metabolic pathway–related enzymes) could be a subtype-independent risk factor in breast cancers, certain lung tumors rhabdomyosarcomas, and pheochromocytomas [128, 207, 334, 335]. In general, mTOR hyperactivity and the expression of mTORC2 complex elements were analyzed and characterized in correlation with prognosis and metastasis of other malignancies. Most of these results confirm the significant role of mTOR hyperactivity in malignant progression.

The site-specific metastatic progression was suggested to be influenced by mTORC1 and mTORC2 activity in liver, lung, bone, brain, colon, breast, and pancreatic cancers or melanomas. The incidence of *PIK3CA* mutations, the activation of PI3K/Akt/mTOR signaling axis, and, in correlation with these, the increased mTORC2 activity (p-Ser473-Akt)–related HER2 expression were found to be involved in the liver metastases of breast cancers, which are occurring more frequently [336]. However, another study emphasized the potential role of PI3K/Akt/mTOR axis hyperactivity in brain metastases of melanomas. mTOR-related hyperactivity correlated with metastases of other cancers was reported in a few lower case number studies (e.g., breast, lung, and renal carcinomas) [334, 337]. There is a strong correlation between high mTOR activity and poor prognosis, significantly increasing mTORC2 complex expression analyzed by IHC follow-up studies.

Regarding these observations, Rictor overexpression results and *RICTOR* amplification were found to negatively influence the progression of different cancers [304]. This could help in patient selection for mTORC1 and mTORC2 or dual inhibitor therapies (Table 2). Several experimental studies described that dual inhibitors could reduce brain metastasis formation and growth of *PI3KCA*-mutated cancers [338]. Additionally, in colon carcinomas and highly aggressive pancreatic cancers, dual mTOR inhibitors could reduce the occurrence of liver and lung/liver metastases, respectively [339].

**Table 2:** The application of PI3K/Akt/mTOR inhibitors in different cancers

Target	Drug name	Tumor type		ID number and the highest developmental stage		Status
**PI3K inhibitors**						
PI3K pan-inhibitor	Copanlisib	Lymphoma				**Marketed**
	Buparlisib (NVP-BKM120)	Head and neck cancer		Phase III — NCT04338399		**Active**
PI3Kα	Alpelisib	Breast cancer				**Marketed**
	Inavolisib (GDC-0077)	Breast cancer		Phase III — NCT04191499		**Active**
	Serabelisib (INK-1117)	Solid tumor		Phase II — NCT04073680		**Active**
	MEN1611 (CH5132799)	Colorectal cancer		Phase II — NCT04495621		**Active**
	CYH-33 (HHCYH-33)	Solid tumor		Phase I — NCT04586335, NCT04856371		**Active**
PI3Kβ	AZD8186	Solid tumor		Phase II — NCT04001569		**Active**
	GSK2636771	Lymphoma, myeloma, solid tumor		Phase II — NCT02465060		**Active**
PI3Kγ	Eganelisib (IPI-549)	Breast cancer, kidney cancer		Phase II — NCT03961698		**Active**
PI3Kδ	Idelalisib	Lymphoma, leukemia				**Marketed**
	Umbralisib	Lymphoma				**Marketed**
	Parsaclisib (INCB50465)	Myelofibrosis, lymphoma		Phase III — NCT04551066, NCT04551053, NCT04796922, NCT04849715		**Active**
	Zandelisib (PWT-143)	Lymphoma		Phase III — NCT04745832		**Active**
	SHC014748 (SH-748)	Lymphoma		Phase II — NCT04470141, NCT04431089		**Active**
	Linperlisib (YY-20394)	Lymphoma		Phase II — NCT04500561, NCT04705090, NCT04370405, NCT04379167, NCT04948788		**Active**
	IOA-244	Lymphoma, melanoma, solid tumor		Phase I — NCT04328844		**Active**
PI3Kδ, PI3Kγ	Duvelisib	Lymphoma, leukemia				**Marketed**
PI3Kδ, PI3Kγ	Tenalisib (RP6530)	Breast cancer		Phase II — NCT05021900		**Active**
PI3Kα, PI3Kδ, PI3Kγ	Taselisib	Solid tumor, lymphoma, myeloma		Phase II — NCT02465060		**Active**
Undisclosed PI3K isoform inhibitor	TQ-B-3525	Lymphoma, leukemia, endometrial cancer, ovarian cancer, breast cancer		Phase II — NCT04615468, NCT04610970, NCT04398953, NCT04324879, NCT04836663, NCT04808570, NCT04355520		**Active**
	HMPL-689	Lymphoma		Phase II — NCT04849351		**Active**
PI3K, mTOR dual inhibitor	Paxalisib	CNS cancer		Phase III — NCT03970447		**Active**
	Samotolisib	Solid tumor, lymphoma		Phase II — NCT03213678, NCT03155620		**Active**
	Gedatolisib	Breast cancer		Phase II — NCT03698383, NCT03911973		**Active**
Various targets	Pictilisib (GDC-0941), Pilaralisib, ZSTK-474, Sonolisib, SAR260301, Seletalisib (UCB-5857), AMG319 (ACP-319), Nemiralisib (GSK2269557), Dezapelisib (NCB-040093), AZD-8835, Dactolisib, Apitolisib, SF1126, Bimiralisib (PQR309), Voxtalisib, etc					**Inactive/discontinued**
**mTOR inhibitors**						
Allosteric mTOR inhibitor	Everolimus	Breast cancer, kidney cancer, endocrine tumor, CNS cancer				**Marketed**
	Sirolimus	Lymphangioleiomyomatosis (LAM)				**Marketed**
	Temsirolimus	Kidney cancer				**Marketed**
mTOR kinase inhibitor	Sapanisertib (MLN0128)	Solid tumor, lymphoma, myeloma		Phase II — NCT02465060		**Active**
	Vistusertib	Lung cancer		Phase II — NCT02664935, NCT03334617		**Active**
	CC-115	CNS cancer		Phase II — NCT02977780		**Active**
	Onatasertib (ATG-008)	Solid tumor		Phase II — NCT04518137, NCT03591965, NCT04998760, NCT04337463		**Active**
PI3K, mTOR dual inhibitor	Paxalisib	CNS cancer		Phase III — NCT03970447		**Active**
	Samotolisib	Solid tumor, lymphoma		Phase II — NCT03213678, NCT03155620		**Active**
	Gedatolisib	Breast cancer		Phase II — NCT03698383, NCT03911973		**Active**
Various targets	Dactolisib, Apitolisib, SF1126, Bimiralisib (PQR309), Voxtalisib, Ridaforolimus (Deforolimus, MK-8669), AZD8055					**Inactive/discontinued**
**Akt inhibitors**						
Pan-Akt	Ipatasertib	Prostate cancer, breast cancer		Phase III — NCT04650581, NCT04177108, NCT04060862, NCT03337724, NCT03072238		**Active**
	Capivasertib	Breast cancer, prostate cancer		Phase III — NCT03997123, NCT04493853, NCT04862663, NCT04305496		**Active**
	Triciribine (PTX-200)	Leukemia		Phase II — NCT02930109		**Active**
	TAS-117	Solid tumor		Phase II — NCT04770246		**Active**
	Afuresertib	Ovarian cancer, prostate cancer		Phase II — NCT04060394, NCT04374630		**Active**
	MK-2206	Lung cancer, thymoma, breast cancer		Phase II — NCT01042379, NCT01306045		**Active**
	Uprosertib	Myeloma, solid tumor		Phase II — NCT01989598, NCT01902173		**Active**
Various targets	COTI-2, Perifosine, LY-2503029					**Inactive/discontinued**

Based on these results and the detected high mTOR activity in many cancers, mTOR inhibitor therapies have been tested in highly metastatic and aggressive cancer types characterized by high mTOR activity (e.g., pancreas, relapsed HER2 + , and TN breast cancers, EGFRI-resistant or cisplatin-, radiotherapy-resistant cancers). mTORIs have been approved in the therapy of renal cancers; mantle cell lymphomas; advanced breast cancers (hormone receptor–positive); pancreatic, gastrointestinal, and pulmonary neuroendocrine cancers; and sporadic lymphangioleiomyomatosis. Despite the correlation between high mTOR activity and worse prognosis, it might be surprising that clinical phase trials usually have lower beneficial therapeutic outcomes; mTORIs also have a lower success rate in monotherapy. Therefore, better mTOR and more specific mTORC1 and mTORC2 inhibitors are needed to be developed. Next-generation mTOR inhibitors include dual mTOR and ATP competitive mTOR kinase inhibitors (2nd generation) [340]. The 3rd-generation new synthetic mTOR (e.g., JR-AB2-011, RapaLinks) and specific mTORC2 complex inhibitors (Rictor si-NPs: nanoparticles harboring Rictor siRNA sequences) are under preclinical and clinical investigations [330, 340]. Additionally, more and more mTOR and PI3K/Akt/mTOR pathways inhibiting natural compounds have been discovered and tested in clinical trials. For example, indole-3-carbinol (an alkaloid from *Brassica genus* — NCT00579332), berberine (an alkaloid from European barberry — NCT03281096, NCT02226185), curcumin (diarylheptanoid from *Curcuma genus* — NCT03769766), epicatechin and other catechins (flavonoids from green tea — NCT02029352), and genistein (isoflavone from soybean — NCT00584532) are in phase III trials, and these have favorable results and are available as dietary supplements. Many different PI3K/Akt/mTOR inhibitors have been under development in clinical trials. These all have promising effects in preclinical studies (Table 3). Unfortunately, the application of mTORI monotherapy has often lower final survival advantages than expected, as is the unfortunate case of targeted monotherapies in general. Therefore, the selection of optimal partner drugs needs to be considered for achieving more effective combination therapies. The combination of mTORIs with targeted or traditional chemotherapeutics/radiotherapy could be applied and initiated in clinical trials (Table 4). However, the possible higher toxicity could limit the success of these combined treatments. Based on the highlighted role of metabolic profiles, other antimetabolic drugs with traditional therapies or mTORIs should be considered to find additional therapeutic options. Some new preclinical studies highlight that mTORIs combined with metabolic (e.g., GLS, OXPHOS, lipid metabolism) inhibitors could have additive or synergistic effects on tumor growth in advanced cancer, in *in vitro* and *in vivo* models [341–343].

**Table 3:** Relevant publications about mTORC2 complex–specific Rictor overexpression in different cancers

Cancer type	Tumor subtype	Rictor overexpression data	Prognostic and therapeutic associations	References
Some latest pan-cancer data	Reviewed genetic alterations (frequent mutations of PI3K/Akt/mTOR pathways in lung, gastric, colorectal, renal, urinary bladder, prostate, and breast cancer and head and neck squamous cell carcinomas) in correlation with mTOR hyperactivity [323]			
	435 cases (colorectal, gastric, renal, hepatocellular, lung cancers, cholangiocarcinoma, sarcomas, and other cancers); Rictor 78% positivity and 29% higher expression (with heterogeneous staining pattern) [344]			
Lung cancer				
	SCLC	6–15% amplification	ns	[344–347]
	Adenocarcinoma	8–10% amplification; 37% overexpression (IHC)	Stage and brain metastasis could correlate	[334, 348]
	Metastatic lung cancers	38% amplification	ns	[349]
	Lymphangioleiomyomatosis	54% overexpression (IHC)	ns	[205]
	Additional importance	*Rictor* amplification–driven therapy has survival benefit in SCLC		[350]
Breast cancer		37–50% overexpression (IHC)	Correlate with worse progression, metastasis Rictor mRNA correlate with worse outcome in patients with basal-like TN, mTOR hyperactivity, and metabolic plasticity correlates with worse prognosis	[128, 314, 318]
	TN	41% overexpression (IHC)		[128, 330]
	HER2 +	58% overexpression (IHC)		
	Luminal A-B	55% overexpression (IHC)		
	Lymph node metastasis	92% overexpression (IHC)		[351]
	Additional results: Rictor is an important mediator of chemotaxis and metastasis in breast cancer cells; *BRCA1* loss is correlate with mTORC2 hyperactivity which can be targeted by mTORC2 inhibition			[352, 353]
Gastrointestinal cancer				
	Gastric	Rictor mRNA, amplification, overexpression (IHC) 50–78% overexpression (IHC)	Rictor overexpression correlates with therapy resistance, worse prognosis, shorter OS; higher levels of Rictor/Akt correlate with poor survival, OS; mTORI combinations could be helpful in both EGFRI and platinum-based therapy of colon carcinomas; high mRNA-independent prognostic indicators for DFS	[349, 354–357]
	Colorectal	Rictor mRNA, overexpression (IHC)		[349, 355, 358, 359]
	Esophageal squamous cell	70% overexpression (IHC)		[360]
	Hepatocellular, cholangiocarcinoma	mRNA		[361–363]
Head and neck cancer		68–90% overexpression (IHC)	Higher stage and invasion	[360, 364–366]
	HPV-related OPSCC	mRNA, overexpression (IHC)	HPV is associated with Rictor overexpression, worse prognosis	[367]
CNS malignancies	Glioma, glioblastoma	64–86% overexpression (IHC)	mTORC2 could be a potential target in therapy	[368–371]
Pancreas cancer		15% overexpression (IHC)	Decreased overexpression and promising results with mTORC1-2 inhibitors, mTORC2 activity marker correlates with worse survival	[372, 373]
Prostate cancer		*In vitro* and other signaling failures data	ns	[372, 374]
Renal cancer			Rapalog resistance in correlation with mTORC2	[375]
Urogenital cancers				
	Endometrial cancer	44% overexpression (IHC)	Correlation with stage, metastasis, prognosis	[376]
	Bladder cancer	*In vitro* data	mTORC2 can mediate bladder cancer cell invasion	[377]
Sarcoma		20% overexpression (mainly leiomyosarcoma) overexpression (IHC)	High mTOR activity is associated with worse prognosis	[378]
	Rhabdomyosarcoma	77% overexpression (IHC), 52% high mTORC2		[207]
	Myxofibrosarcoma	42% amplification		[379]
Other cancers				
	Lymphomas/leukemias	DLBCL (non-GC), AML, ALL, CLL, MCL, mRNA, experimental data, overexpression (IHC)	Higher mTORC2 activity correlates with worse prognosis, mTORC2 inhibition promising	[325, 380–385]
	Melanoma	mRNA	Liver metastasis in correlation with high Rictor expression; Rictor inhibition decrease metastasis	[386, 387]
	Pheochromocytoma	80% overexpression (IHC)	ns	[388]

**Table 4:** Ongoing combination therapy with mTOR inhibitors in different cancers

Tumor type	Ongoing combination trial		
	**Intervention/treatment**	**Study ID number**	**Phase**
**Breast**			
Marketed: metastatic, HR + /HER2 − breast cancer (Everolimus)			
**Luminal A (HR + /HER −)**	AZD2014/Everolimus + Fulvestrant	NCT02216786	Phase II
	Everolimus + Paclitaxel	NCT04355858	Phase II
**HER + (HR − /HER +)**	Rapamycin + Inetetamab + Chemotherapy	NCT04736589	Phase III
**TN**	AZD2014 + AZD6244 (MEKI)	NCT02583542	Phase I/II
	Everolimus + Bevacizumab + Doxorubicin	NCT02456857	Phase II
**Others**	Temsirolimus + AZD6244	NCT00600496	Phase I
**Colorectal**			
	Nab-rapamycin + FOLFOX6 + Bevacizumab	NCT03439462	Phase I/II
	Temsirolimus + AZD6244	NCT00600496	Phase I
**Lung**			
Marketed: sporadic lymphangioleiomyomatosis (rapamycin)			
**NSCLC**	Gedatolisib + Palbociclib	NCT03065062	Phase I
	Rapamycin + Epacadostat	NCT03217669	Phase I
	AZD2014 + AZD6244 (MEKI)	NCT02583542	Phase I/II
	Sirolimus + Durvalumab	NCT04348292	Phase I
**NSCLC + SCLC**	Rapamycin + Auranofin	NCT01737502	Phase I/II
	Temsirolimus + AZD6244	NCT00600496	Phase I
**Head and neck**			
	Gedatolisib + Palbociclib	NCT03065062	Phase I
**Urogenital**			
	Temsirolimus + Paclitaxel + Carboplatin	NCT00977574	Phase II
	AZD2014 + Anastrozol	NCT02730923	Phase I/II
	Rapamycin + Auranofin	NCT03456700	Phase II
	Everolimus + Levonorgestrel	NCT02397083	Phase II
	Everolimus + Letrozole + Ribociclib	NCT03008408	Phase II
	ATG008/ATG010	NCT04998760	Phase II
**Leukemia**			
	Decitabine + Rapamycin	NCT02109744	Phase I/II
	Rapamycin + Azacitidine	NCT01869114	Phase II
	Rapamycin + Tacrolimus + Melphalan + Clofarabine	NCT01885689	Phase II
**Lymphoma**			
Marketed: mantle cell lymphoma (temsirolimus)			
**Hodgkin**	Everolimus + Itacitinib	NCT03697408	Phase I/II
**Pancreatic**			
	Gedatolisib + Palbociclib	NCT03065062	Phase I
**Renal**			
Marketed: metastatic renal cancer (everolimus, temsirolimus)			
	Temsirolimus + Sunitinib	NCT01517243	Phase II
	Temsirolimus + AZD6244	NCT00600496	Phase I
	Everolimus + DFF332 (HIF2αI)	NCT04895748	Phase I
	Everolimus + Lenvatinib	NCT03324373	Phase I
	Everolimus + Lenvatinib	NCT05012371	Phase II
**Brain, CNS**			
Marketed: astrocytoma (everolimus)			
	Temsirolimus + Perifosine	NCT02238496	Phase I
	Temsirolimus + Vorinostat	NCT02420613	Phase I
	Nab-rapamycin + Temozolomide + Irinotecan	NCT02975882	Phase I
	Temsirolimus + Dasatinib + Cyclophosphamide	NCT02389309	Phase I
	Nab-rapamycin + standard therapy	NCT03463265	Phase I
	Sirolimus + Celecoxib + Etoposide + Cyclophosphamide	NCT02574728	Phase II
	Everolimus + Trametinib	NCT04485559	Phase I
**Melanoma**			
	Temsirolimus + AZD6244	NCT00600496	Phase I
**Sarcoma**			
	Nab-rapamycin + Pazopanib hydrochloride	NCT03660930	Phase I/II
	Temsirolimus + chemotherapy	NCT02567435	Phase III
	Everolimus + Ribociclib	NCT03114527	Phase II
	Everolimus + Temsirolimus + other chemotherapeutic drugs in microdose in implanted percutaneous microdevice	NCT04199026	Early phase I
**Neuroendocrine**			
Marketed: neuroendocrine tumors originating in the lungs or gut, pancreatic neuroendocrine tumors (everolimus)			
	Everolimus + Lenvatinib	NCT03950609	Phase II
	Everolimus + Bevacizumab + Octreotide acetate	NCT01229943	Phase II
**Others**			
**Advanced cancer**	Rapamycin/Everolimus/Temsirolimus + Vorinostat	NCT01087554	Phase I
	Temsirolimus + Bevacizumab + Carboplatin/Sorafenib/Paclitaxel	NCT01187199	Phase I
	Everolimus + Vandetanib	NCT01582191	Phase I
	Rapamycin/Everolimus + Cemiplimab + Prednisone	NCT04339062	Phase I
	Everolimus + Ceritinib	NCT02321501	Phase I
**Solid tumor**	Temsirolimus + Ixabepilone	NCT01375829	Phase I
	Nab-rapamycin + Temozolomide + Irinotecan	NCT02975882	Phase I
	Temsirolimus + Dasatinib + Cyclophosphamide	NCT02389309	Phase I
	Gedatolisib + Palbociclib	NCT03065062	Phase I
	Temsirolimus + Valproic acid + Cyclophosphamide + Bevacizumab	NCT02446431	Early phase I
	Everolimus + Trametinib + Lenvatinib	NCT04803318	Phase II
	Rapamycin + Epacadostat	NCT03217669	Phase I
	Sirolimus + Celecoxib + Etoposide + Cyclophosphamide	NCT02574728	Phase II
**Neurofibromatosis**	Rapamycin + Selumetinib (MEKI)	NCT03433183	Phase II
	Rapamycin + PLX3397 (MTKI)	NCT02584647	Phase I/II
**Neuroblastoma**	Temsirolimus + Temozolomide + Irinotecan	NCT01767194	Phase II
**Hepatoblastoma**	Temsirolimus + chemotherapy	NCT00980460	Phase III
**Hepatocellular cc**	Everolimus + Trametinib + Lenvatinib	NCT04803318	Phase II
**Vascular tumor**	Rapamycin + Prednisolone	NCT03188068	Phase II

Such combined antimetabolic treatments can induce synthetic lethality [389]. For example, inhibiting both OXPHOS and glycolysis (e.g., with metformin + 2-deoxy-D-glucose/GLUT/MCTI) can effectively induce the collapse of balanced energy metabolism, which was detected to be effective in many different cell line and xenograft models (e.g., breast, prostate, ovarian, and hepatocellular cancers) [159, 390, 391]. In the case tumors depend on glutamine utilization and anaplerosis, this metabolic rewiring provides a potential opportunity for rational dual targeting of glutaminolysis and glycolysis. Simultaneous glutaminolysis blockade with CB-839 and 3-bromopyruvate (3BP) could also induce tumor regression in the renal cancer model [392]. These preclinical combination studies initiated a phase I study using ritonavir and metformin in the management of multiple myeloma and chronic lymphocytic leukemia (CLL) (NCT02948283). Further preclinical data and key clinical trials using metabolic inhibitors are available; including mTORIs combined with conventional tumor type–dependent and targeted therapy (including immune checkpoint inhibitor therapy) [393]. There are several promising preclinical observations regarding the potential use of antimetabolic (OXPHOS or glutamine metabolism inhibitors) and targeted kinase inhibitory therapy in various cancer types (e.g., melanoma, GIST, etc.) (NCT03026517, NCT03831932) [394–396]. These have propagated further clinical studies (lung RCC NCT03158324, NCT03071705, and NCT02071862). According to this promising preclinical data, early-phase trials of rapalog and metformin combinations were set off. These suggest that further examinations of temsirolimus and metformin is necessary since this combination reported good tolerance and promising effects in advanced cancers (NCT01529593, NCT03163667, and NCT00659568). Moreover, immune checkpoint inhibitors or other monoclonal antibody therapies promoted the attempts of glutamine utilization inhibitor (including mTORIs) combinations with pembrolizumab, nivolumab, or daratumumab in advanced solid cancers (NCT04265534 and NCT02771626) [397]. Other agents and drugs (e.g., antibiotics, cardiovascular drugs, antidepressants) [398] with antimetabolic (off-target) properties can be applied in an off-label use in these combinations in future studies [399–401].

All these data suggest that there is an urgent need to clarify the role of metabolic adaptations in tumor resistance, with respect to recently used therapies (metabolic rewiring at tissue level). Future implications in this field of research are to expand the clinically available metabolic targets (drugs) to improve the response rate and patient survival in recently used treatments.

## Concluding remarks

*Albert Szent-Györgyi* (who won the Nobel Prize for discovering the components and reactions in TCA in 1937 and isolated vitamin C) said — “Nature is huge, man is tiny. Therefore, man's existence depends on what kind of interactions he can make with nature, how much he understands it, and how he uses its resources for his benefit.” In the last decades, we have discovered many various mechanisms and their alterations, complexity, and tumor type dependency in the genetic, epigenetic, and metabolic deregulation of tumor growth. Now, we have ideas how tumors reprogram their proliferation, survival mechanisms, and the microenvironment, the organ, the ecosystem, and even the whole body. Finally, we understand that “*Man* is huge, *the tumor cell* is tiny. Therefore, *tumor’s* existence depends on what kind of interactions it can make with the *environment*, how much *tumor* “understands” this, and how it uses the environmental resources for its benefit.” The complexity of heterogeneous tumors, including different cell populations and their symbiosis, further complicates our understanding of how tumors can develop. Consequently, the local conditions are altering (hypoxic, acidic, and nutrient-deprived environment) and promoting metastasis (“escape”) to find another niche to grow and invade the whole body and use its sources. Finally, the metabolic plasticity and all the related forces in the human body serve the survival of the tumor population. During this, the tumor disrupts the homeostasis of the host, which leads to the death of the patient. After the scientific revolution in different research fields, primarily due to oncogenomic and molecular carcinogenesis studies, we could describe and comprehend many pieces of carcinogenesis and progression. However, to overcome cancer therapy failures, we need to understand oncogenic networks, including the regulatory shortcomings of metabolic adaptation in this complex and dynamic cancer ecosystem.
